# Initial data release of regular blood drip stain created by varying fall height, angle of impact and source dimension

**DOI:** 10.1016/j.dib.2016.07.003

**Published:** 2016-07-06

**Authors:** Nabanita Basu, Samir Kumar Bandyopadhyay

**Affiliations:** University of Calcutta, India

**Keywords:** Drip stain, Bloodstain Pattern Analysis, Source Dimension prediction

## Abstract

The dataset developed consists of 108 blood drip stains developed with fresh porcine blood, blood admixed with different dosage of Warfarin and Heparin, respectively. For each particular blood type (i.e. fresh blood, blood admixed with Warfarin at different dosage and blood admixed with Heparin at varied dosage) stain patterns were created by passive dripping of blood from a 2.5 cm^3^ subcutaneous syringe with needle filled to capacity, at 30°, 60° and 90° angle of impact with corresponding fall height of 20, 40 and 60 cm respectively. In the other dataset of 162 datapoints, 81 regular drip stains were formed from blood that had dripped passively from a subcutaneous syringe without needle at the aforementioned angle of impact and fall height, while the other stains were formed as a result of dripping of blood from a subcutaneous syringe with needle. In order to compare stains formed, all stains were recorded on the same representative, non-porous, smooth target surface under similar physical conditions. The interpretations relevant to the dataset are available in the article titled ‘2D Source Area prediction based on physical characteristics of a regular, passive blood drip stain’ (Basu and Bandyopadhyay, 2016) [7]. An image pre-processing algorithm for extracting ROI has also been incorporated in this article.

## Specifications Table

**Table:** 

Subject area	*Statistics, Computer Science*
More specific subject area	*Machine Learning*
Type of data	*Figure, table*
How data was acquired	*Data was acquired with Camera, Nikon COOLPIX L610*
Data format	*Raw data*
Experimental factors	*Atmospheric Conditions [Dry Temperature: 23 *^*o*^*C, Wet Temperature: 26 *^*o*^*C, Relative Humidity-77–78%, Wind Condition: Not Windy], Blood Temperature: 37 *^*o*^*C. Experimental Room[ Temperature: 37* ^*o*^*C, Humidity: 60%, Wind Condition:Not Windy]*
Experimental features	*The experiment was undertaken at the Kolkata Municipal Slaughter House.*
	*Pig blood was allowed to drip from a subcutaneous syringe filled to capacity (2.5* *cc) under the action of gravity.*
	*Blood was allowed to drip from a height of 20, 40 and 60 cm at 30°, 60° and 90° respectively.*
	*All stains were taken on 75 GSM white paper and allowed to dry before photography.*
Data source location	*Kolkata Pig Slaughter House, Tangra, Kolkata, West Bengal, India*
	*Latitude: 22.5577172; Longitude: 88.38665859999992*
Data accessibility	*Data described is within this article and is available at Figshare*
	https://figshare.com/s/4207047f45a4add5fd76

## Value of the data

● The dataset is the first of its kind.
● No dataset for bloodstain patterns is available as a benchmark dataset for computer scientists or mathematicians who do not have access to resources to create such a database.
● The dataset can be processed in a standard computer with 4 GB RAM.
● The dataset has been developed as per the physical evidence documentation guidelines followed in Forensic science.

## 1 Data

Passive drip stains were developed from blood droplets of fresh porcine blood, blood thoroughly admixed with different dosage of Warfarin (i.e. 2 mg, 4 mg, 6 mg, 8 mg, 10 mg) and Heparin (i.e. 260 I.U., 520 I.U., 780 I.U., 1040 I.U., 1300 I.U.) emanated from a subcutaneous syringe with and without needle by varying the angle of impact in the range of 30°, 60° and 90° against fall height of 20, 40 and 60 cm [1], [2] respectively. The drip stains were photographed based on the guidelines followed for documentation of physical evidence in forensic science. Table 1 documents the standards used for archiving the drip stain pattern images.

## 2 Experimental design, materials and methods

Pig blood was used in developing the bloodstain dataset as pig blood rheology closely resembles human blood rheology [3], [4]. The experiment was undertaken at the Kolkata Municipal Pig Slaughter House with due permission from the concerned authorities.

All experiments that were conducted with fresh porcine blood were performed within 15 min from the time of collection of blood from a freshly slaughtered pig. For experiments in which blood thoroughly admixed with Warfarin was used, the container in which blood was collected was lined with aqueous solution of Warfarin prepared with distilled water. Again, experiments in which blood thoroughly admixed with Heparin was used, the container in which blood was collected was lined with different dosage of Heparin based on experimental requirements. Blood admixed with Warfarin or Heparin was allowed to stand for 24 h in an antechamber at the slaughter house that was maintained at 4 °C with stirring by an automatic overhead stirrer at 20 rpm [5]. Two hours prior to experimentation, blood admixed with Warfarin or Heparin was placed in a chamber maintained at 37 °C with light stirring (20 rpm) by an automated overhead stirrer. Experimentation was started only when the temperature of blood was found to be 37 °C on a digital thermometer.

**Fig. 1:**
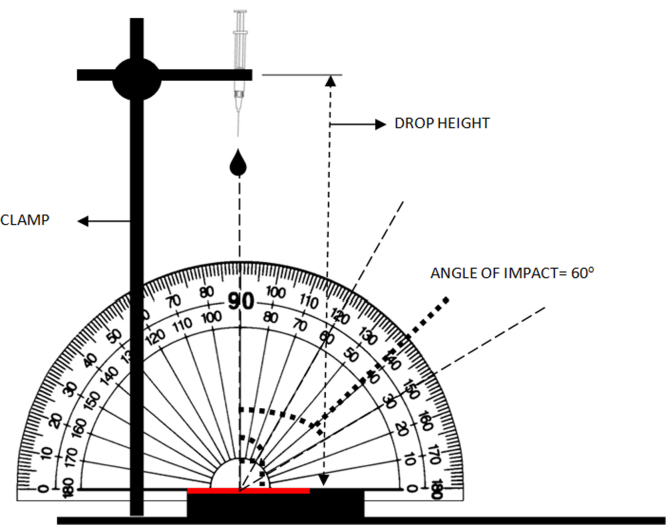
Experimental setup for creating stain patterns at different angles of impact and fall height. The dotted lines along 30° and 60° represent the position of the target surface for an impact angle of 60° and 30° respectively.

The experiment was performed in 2 parts. The first part dealt with development of drip stains from blood drops emanated from a subcutaneous syringe with needle. The angle of impact and the fall height were varied. Fig. 1 represents the experimental setup. The subcutaneous syringe was clamped and a protractor was attached to the clamp as demonstrated in Fig. 1.

In order to achieve a particular angle of impact, the angle of inclination of the target surface was varied. A centimeter scale was used to calibrate fall height. Pressure required to eject a single blood droplet pendant from the subcutaneous syringe with needle was empirically calculated. Table 2 represents the angle of impact and fall height combination set used for the development of drip stain with fresh porcine blood, 250 ml of blood thoroughly admixed with different dosage of Warfarin and 250 ml of blood thoroughly admixed with different dosage of Heparin.

In the second part of the experiment, the experimental setup illustrated in Fig. 1 was used to develop drip stain patterns at varying angle of impact and fall height by allowing blood pendant formation and subsequent dripping under the action of gravity from a subcutaneous syringe with and without needle. Table 3 documents the specification of impact angle and fall height used for development of drip stain patterns in the second part of the experiment.

All of the experiments were carried out under similar environmental conditions. The clean room in which the experiments were performed was maintained at 37° C. Table 4 represents the atmospheric conditions maintained within the laboratory across all experiments undertaken.

During each dripping done, the column of blood in the syringe was maintained at 2.5 cc with no air bubbles. In order to compare the drip stains formed, all stains were recorded on the same representative non-absorbent/non-porous, smooth paper target surface. Table 5 provides the specification of the target surface that has been used for the experiments.

Blood is an integral component in the experiments undertaken. Blood from healthy pigs was used in dataset creation. Table 6 provides a summary of the properties of porcine blood that was used in the experiments. Only singular, non-overlapping, regular drip stains were included in the dataset.

The samples created during the experiment were allowed to dry and hence photographed using a Nikon Coolpix L610 camera. The images were taken by placing horizontal and vertical scales with respect to the stain [6]. The camera lens was held parallel to the target surface on which the stains were formed [6]. The aspect ratio for each image was maintained.

**Fig. 2:**
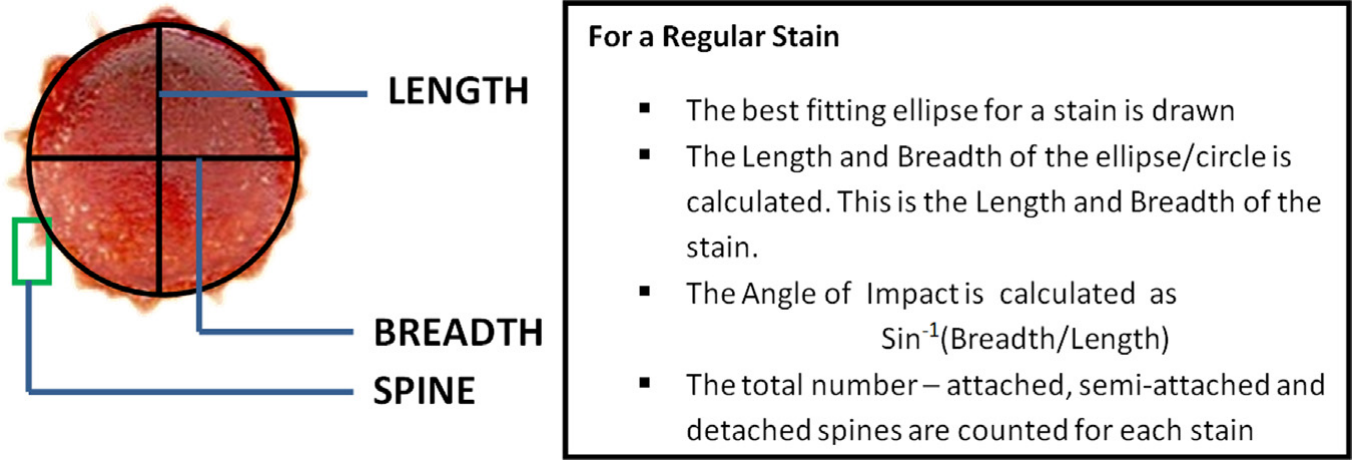
The image represents the method adopted and widely accepted by bloodstain pattern analysts for measuring the length, breadth, angle of impact and total number of spines for each individual stain.

Fig. 2 represents how length, breadth, angle of impact and total number of spines were calculated for each stain pattern.

## 3 Technical validation

Each sample was carefully created. For each particular angle of impact at which the stains were created, the angle of impact was calculated from the static stains using the accepted formula, sin^−1^(breadth/length) [6]. To validate and also to minimize experimental error the following steps were undertaken.

1. Each stain pattern documented in Sheet 1 and Sheet 2 was recreated nine times to minimize error.
2. The dataset in Sheet 2 was shuffled and cross-validated to prevent over-fitting of the prediction model.

The standard deviation and standard error values for the measured parameters, such as length, breadth, total number of spines and calculated angle of impact for the 108 datapoints strong dataset was calculated. The standard error and variance of the samples created with Heparin was estimated. The paper on ‘2D Source area prediction based on physical characteristics of a regular, passive blood drip stain’ reports the length, breadth of stain created using fresh blood and Warfarin were found to follow normal distribution while the length and breadth of stains created with Heparin were found to follow a non-normal distribution [7]. Again, the distribution of the total number of spines for the stains created with Heparin, Warfarin and fresh blood was found to deviate from a normal distribution [7].

**Fig. 3:**
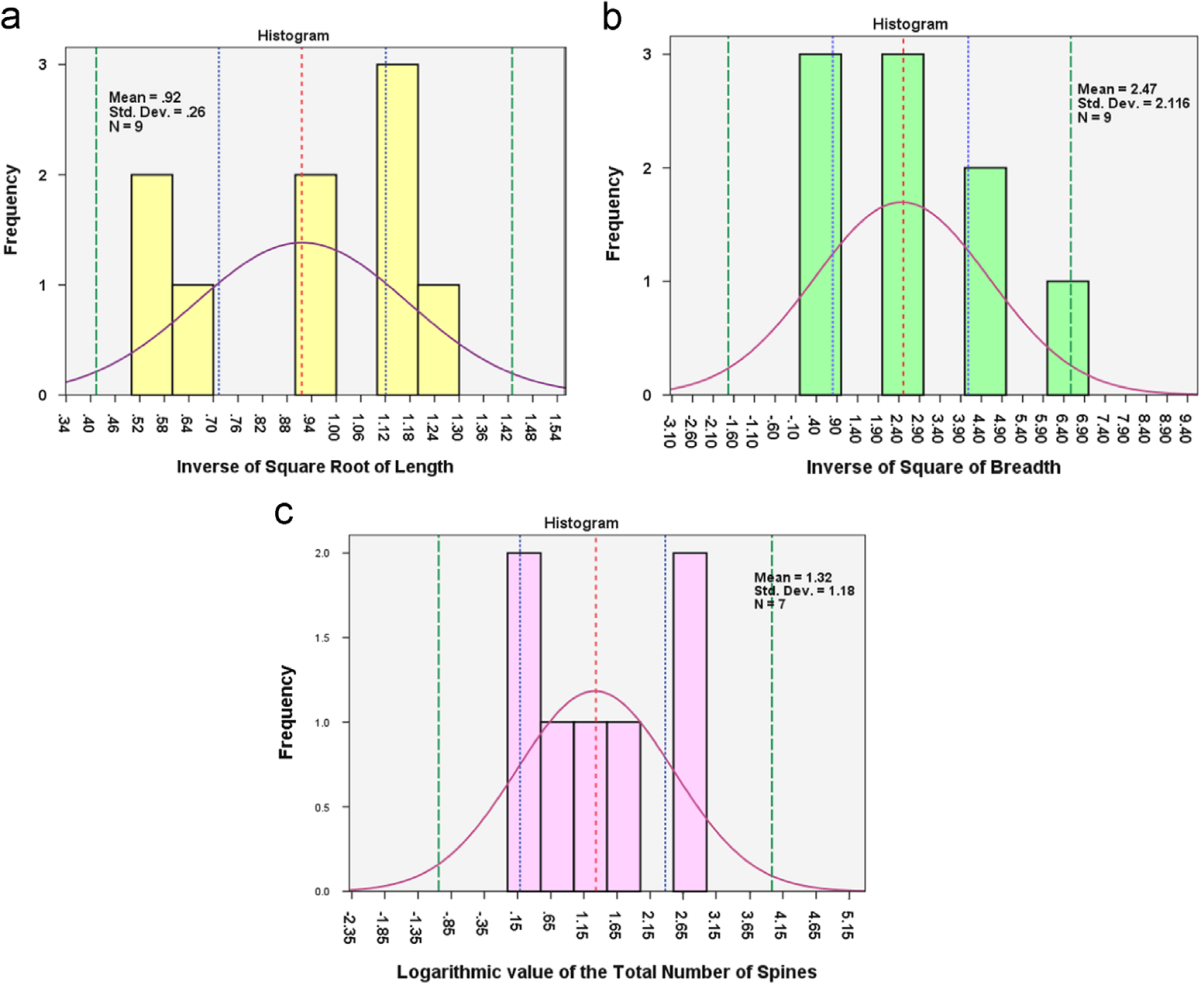
(a) Histogram of the inverse of the square root of length parameter for nine stains created at 30°, 60° and 90° angle of impact with fall height of 20, 40 and 60 cm using a needle filled to capacity with blood (250 ml) thoroughly admixed with 260 I.U. of Heparin [Mean-0.92, S.D.-0.26]. (b) Histogram of the inverse of the square of the breadth parameter for the same stain pattern [Mean-2.47, S.D.-2.116] (c) Histogram of the logarithmic value of the total number of spines associated with each of nine stain pattern created using the same mechanism [Mean-1.32, S.D.-.1.18] [Only 7 points considered as 2 stains recorded absence of any spines]. The curve represents the best fitting normal distribution curve for each of the histograms. The red dotted line represents the mean of the transformed length, breadth and total number of spine values for the nine stains considered. The blue dotted line on either side of the red line represents the lower and upper limit for 95% confidence interval of the mean. The green line on either side represents the range within which 95% of the values lie [For xls Sheet 1 in the Dataset].

The length, breadth and total number of spines of the dataset of Heparin for dosage of 260 I.U. mixed with 250 ml of porcine blood consisting of 9 datapoints can be represented as a normal distribution by using the inverse of the square root of length value, inverse of the square of the breadth and logarithm of the total number of spines as a variable (refer Fig. 3) [8].

The transformed length, breadth and total number of spines for the nine datapoints were found to follow normal distribution with Shapiro Wilk value of 0.862(*p*=0.100>0.05), 0.909(*p*=0.310>0.05) and 0.901 (*p*=0.334>0.05) [df=7, as the number of spines for two of nine datapoints was recorded as zero] respectively. Blood drops were ejected from a 2.5 cc subcutaneous syringe with needle filled to capacity with 250 ml of porcine blood thoroughly admixed with 260 I.U. of Heparin. The points that include 95% of the inverse of the square root of length observations are 0.9164 (mean)±1.96×0.25953(standard deviation), giving a range of 0.4077212 and 1.4250788 [9]. There stands only 5% chance that the population mean will lie outside the points defined by 0.9164±2.306×0.08651 (standard error) [*t*_α/2,df_= 2.306 (df=8)] [9], giving a range of 0.7169 and 1.1159. Similarly, only 5% of the inverse of the square of the breadth observations are expected to lie outside the range 2.4698±1.96×2.11568, defined by the points, −1.67693 and 6.61653. There is 95% chance that the mean of the breadth value of the population will lie in the range defined by the points 2.4698±2.306×0.70523, giving the range values of 0.84354 and 4.09606. The points that include 95% of the logarithm value of the total number of spines are 1.3157(mean)±1.96×1.17956(standard deviation), giving a lower limit value of −0.99624 and upper limit value of 3.62764. There exist only 0.05 probability that the mean of the population of the logarithmic value of the total number of spines will lie outside the range defined by the points 1.3157(mean)±2.306×0.44583 [Lower bound: 0.28762; Upper bound: 2.343784].

**Fig. 4:**
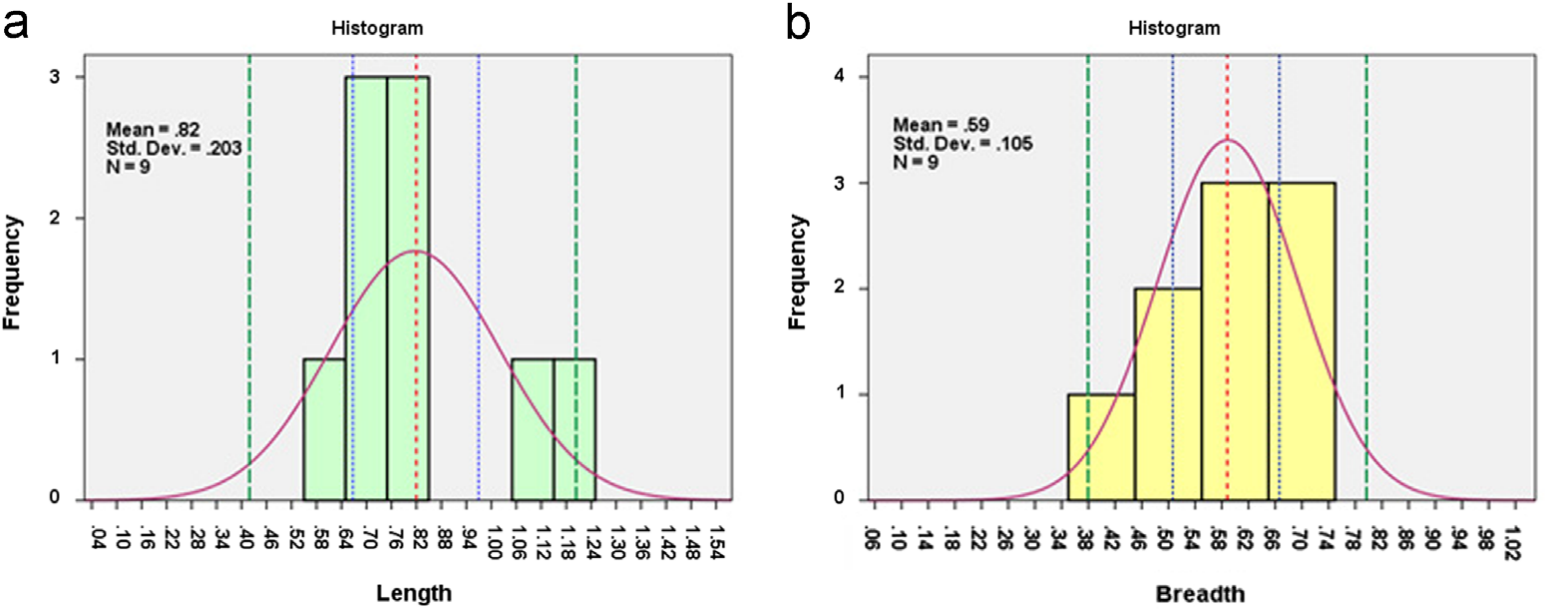
(a) Histogram for the original length value of stains grouped on the basis of source of emission (subcutaneous syringe with needle) and dosage of Heparin (260 I.U.) [mean-0.82, S.D.-0.203]. Consists of stains created at 30°, 60° and 90° angle of impact with fall height of 20, 40 and 60 cm with blood drop ejected from a 2.5 cc subcutaneous syringe with needle filled to capacity with blood thoroughly admixed with 260 I.U. of Heparin. (b) Histogram for the original breadth value for the same stains [Mean-0.59, S.D.-0.105]. The red dotted line represents the mean value for the distribution. The blue lines represent the range within which there is a 95% chance that the population mean will lie. The green line represents the range within which 95% of the datapoints are expected to lie.

Multiple checks on the 108 datapoint strong dataset revealed that certain values of length and breadth in the dataset created with Heparin (dosage – 260 I.U. in 250 ml of porcine blood) had values incorporated from a different scale of measurement. Given that a set of values came from a different measurement scale, these values contributed to the non-normal distribution of the length and breadth parameters. Fig. 4 presents the length, breadth distribution with the actual values by replacing the erroneous ones. The length and breadth values for 9 datapoints created with a 2.5 cc subcutaneous syringe with needle containing blood (250 ml blood thoroughly admixed with 260 I.U. of Heparin), were found to follow normal distribution with Shapiro Wilk value of 0.846 (*p*=0.068>0.05) and .889 (*p*=0.194>0.05) respectively.

**Fig. 5:**
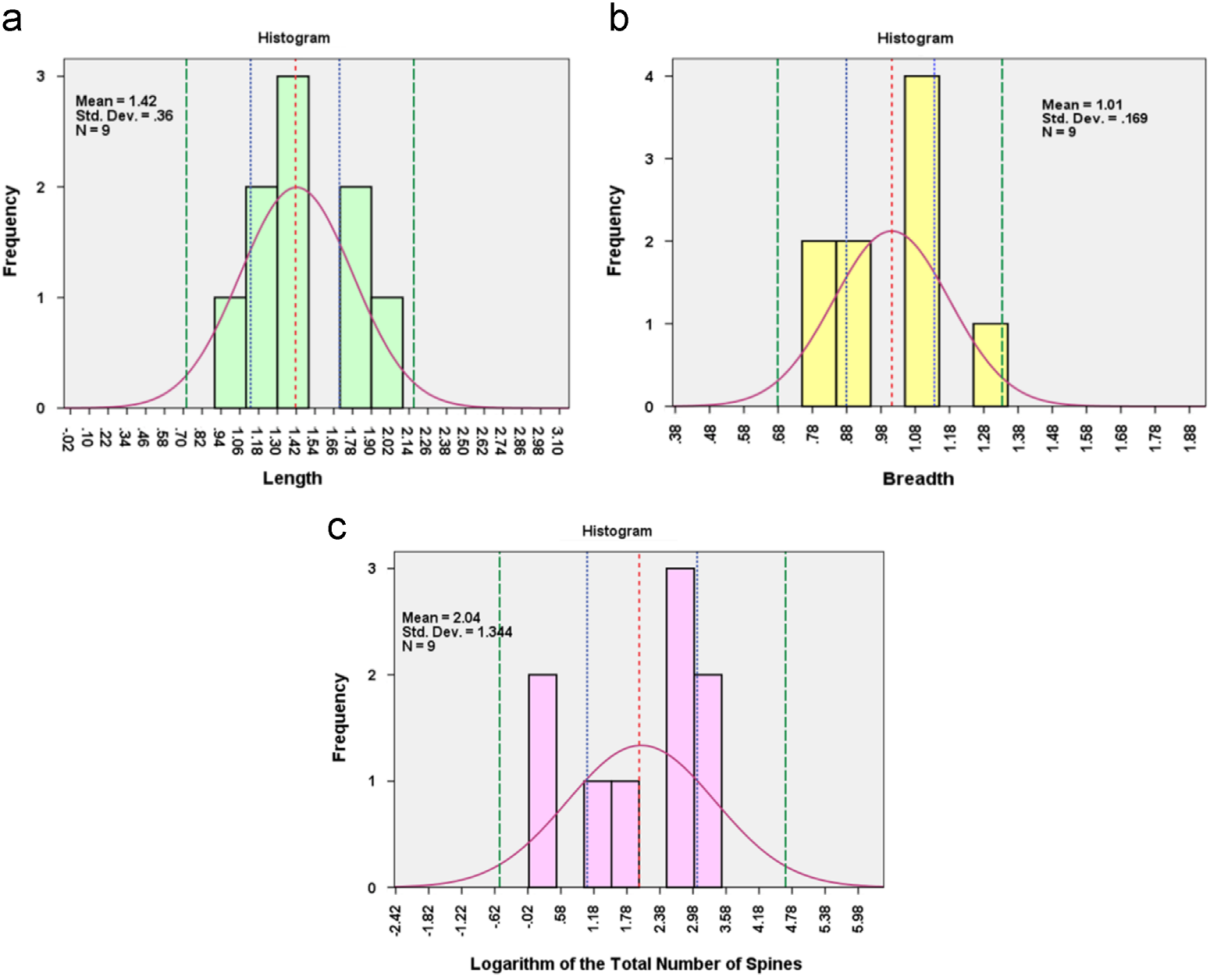
(a) Histogram of the length of the 9 stains grouped on the basis of source of emission (subcutaneous syringe without needle) and dosage (2 mg of Warfarin in 250 ml of blood) taken across all the impact angles (30°, 60° and 90°) and fall height (20, 40 and 60 cm) [Mean-1.42, S.D.-0.36] (b) Histogram of the breadth of the same stains [Mean-1.01, S.D.-0.169] (c) Histogram of the logarithm of the total number of spines for the same stains [Mean-2.04, S.D.−1.344]. The red dotted line represents the mean value for the distribution. The blue lines represent the range within which there is a 95% chance for the population mean to lie. The green line represents the range within which 95% of the datapoints lie. [For xls Sheet 2 in the dataset] [The stain datapoints from subcutaneous syringe without needle were shuffled and a datapoint representative of each impact angle and corresponding fall height was randomly selected.].

The features length, breadth and logarithm of the total number of spines for stains, formed by letting blood thoroughly admixed with 2 mg of Warfarin to drip from a 2.5 cc subcutaneous syringe without needle filled to capacity, were found to follow normal distribution with Shapiro Wilk values of 0.897 (*p*=0.236>0.05), 0.886 (*p*=0.180>0.05) and .838 (*p*=.055>0.05) respectively. The points that include 95% of the length observations are 1.422 (mean)±1.96×0.35978 (standard deviation), giving a range of 0.7170312 and 2.1273688 [based on Empirical Rule]. There stands only 5% chance that the population mean will lie outside the points defined by 1.422±2.306×11,993 (standard error) [*t*_α/2,df_= 2.306 (df=8)], giving a range of 1.14544 and 1.69856. The points that include 95% of the breadth observations for the stains are 1.0111 (mean)±1.96×0.16915 (standard deviation), giving a range of 0.679566 and 1.342643. There stands only 5% chance that the population mean will lie outside the points defined by 1.0111±2.30×0.05638 (standard error) [*t*_α/2,df_= 2.306 (df=8)], giving a range of 0.88109 and 1.141112. The points that include 95% of the logarithm of the total number of spines for the stains formed for the nine stains under consideration are 2.04±1.96×1.34371, giving a range of 0 and 4.674, given that the number of spines cannot be negative. Again, there is 95% chance that the mean of the total number of spines for the entire population will lie in the range 2.04±2.30×44,790, defined by the points 1.0058 and 3.0716 (refer Fig. 5).

When the angle of impact and fall height is taken into consideration in accordance with the source of emission and dosage when grouping stain patterns, the standard deviation from the mean and the median absolute deviation were found to be much lower than the standard deviation value for the length, breadth and total number of spines for stains grouped only on the basis of source of emission and dosage. For Sheet 2 in the dataset, the mean and standard deviation of length, breadth and total number of spines for 9 stains grouped on the basis of dosage (2 mg of Warfarin in 250 ml of blood), emission point (2.5 cc subcutaneous syringe without needle), fall height (40 cm) and angle of impact (60°) are represented in Table 7.

The standard deviation of the calculated angle of impact from the intended angle of impact irrespective of other specification was calculated for the 108 datapoint strong dataset. The variance and standard deviation of the calculated angle of impact from the median value of 30° across 36 datapoints was calculated as 4.8322 degree^2^ and 2.1982° respectively. The standard deviation of the calculated angle of impact from the intended angle of impact (60°) and median value of 59° was recorded as 5.6470° and 5.1695° respectively. For 90° angle of impact the standard deviation and variance was recorded as 0°.

For the dataset represented in ‘Sheet 2’, 9-fold cross-validation was performed to control or prevent over-fitting of the prediction data model.

## 4 Usage hints

**Fig. 6:**
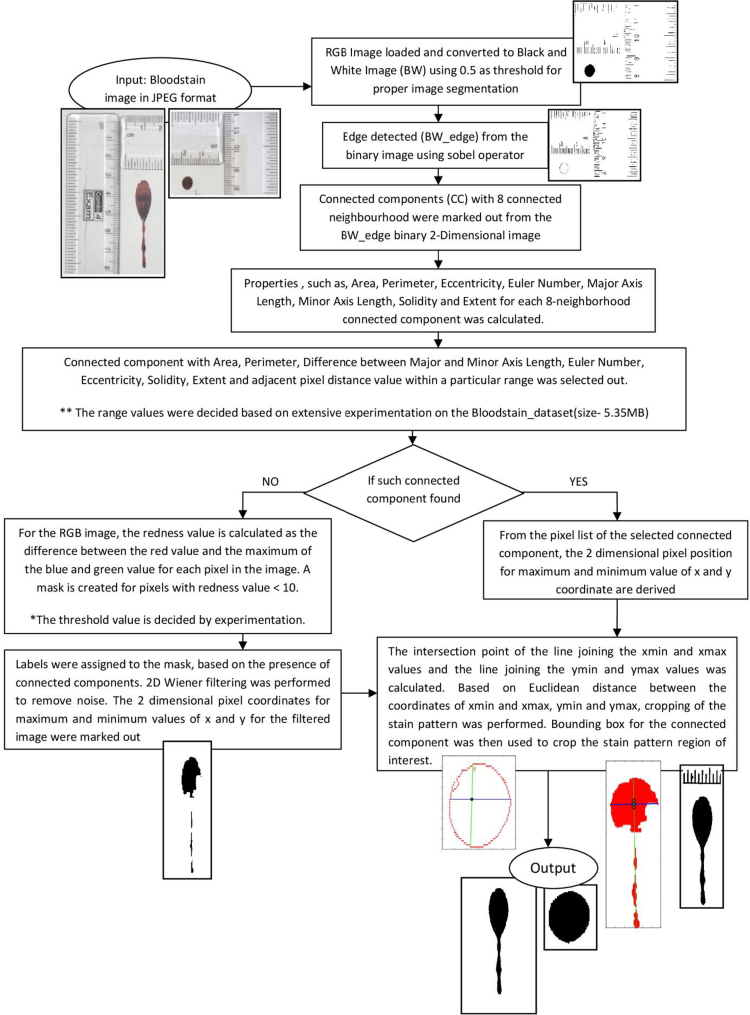
Block diagram of the algorithm used for detecting the Region of Interest (ROI) from the bloodstain pattern images of the dataset ‘Bloodstain dataset’. [The code was developed and executed in MATLAB v7.9.0 (R2009b)].

Image pre-processing is an integral step towards removing noise, artefacts (such as ruler, pencil markings in this case etc.) from an image. Extraction of the Region of Interest (ROI) from an image is the first step towards selection or extraction of relevant features from the image. In order to aid users, the authors have developed an algorithm for pre-processing and thereby extraction of Region of Interest from the images documented in Sheet 1 and 2. Fig. 6 represents a block diagram of the algorithm that might be used for pre-processing and ROI extraction from the image. The time complexity of the algorithm presented is O(n^2^).

## Figures and Tables

**Table 1:** Camera specification used for recording the image of individual drip stain patterns.

**Image specifications**	
IMAGE SIZE	500×760 pixel^2^ (1.667 in ×2.533 in)
IMAGE RESOLUTION	300 dpi
IMAGE FORMAT	.jpg
CAMERA USED	Nikon Coolpix L610
F-NUMBER	F5.1
EXPOSURE TIME	1/15”
FOCAL LENGTH	43.4 mm(equiv. 241 mm on 35 mm film)
ISO SPEED RATING	ISO 400/27°
PROGRAM	NORMAL PROGRAM
MAX. LENS APERTURE	F3.2
COLOR SPACE	sRGB

**Table 2:** Specification of the drip stains developed in the first part of the experiment.

**Blood types**	**Angle of impact (deg.)**	**Fall height (cm)**	**Number of repetitions/Number of drip stains**
Fresh porcine blood	30	20	2
	30	40	2
	30	60	2
	60	20	2
	60	40	2
	60	60	2
	90	20	2
	90	40	2
	90	60	2
Warfarin (250 ml of blood admixed with 2 mg, 4 mg, 6 mg, 8 mg and 10 mg of Warfarin)	30	20	1 for each dosage; total 5
	30	40	1 for each dosage; total 5
	30	60	1 for each dosage; total 5
	60	20	1 for each dosage; total 5
	60	40	1 for each dosage; total 5
	60	60	1 for each dosage; total 5
	90	20	1 for each dosage; total 5
	90	40	1 for each dosage; total 5
	90	60	1 for each dosage; total 5
Heparin(250 ml of blood admixed with 260 I.U., 520 I.U., 780 I.U., 1040 I.U. and 1300 I.U. of Heparin)	30	20	1 for each dosage; total 5
	30	40	1 for each dosage; total 5
	30	60	1 for each dosage; total 5
	60	20	1 for each dosage; total 5
	60	40	1 for each dosage; total 5
	60	60	1 for each dosage; total 5
	90	20	1 for each dosage; total 5
	90	40	1 for each dosage; total 5
	90	60	1 for each dosage; total 5
**Total number of drip stains developed**			**108(18+45+45)**

**Table 3:** The combination of the angle of impact and fall height that have been used for formation of drip stains from a subcutaneous syringe with and without needle when the 2.5 cc subcutaneous syringe was filled to capacity.

**Source aperture**	**Blood type**	**Angle of impact (deg.)**	**Fall height (cm)**	**Number of repetitions/Number of drip stains**
Subcutaneous syringe with needle	250 ml of porcine blood thoroughly admixed with 2 mg of Warfarin	30	20	9
		30	40	9
		30	60	9
		60	20	9
		60	40	9
		60	60	9
		90	20	9
		90	40	9
		90	60	9
Subcutaneous syringe without needle	250 ml of porcine blood thoroughly admixed with 2 mg of Warfarin	30	20	9
		30	40	9
		30	60	9
		60	20	9
		60	40	9
		60	60	9
		90	20	9
		90	40	9
		90	60	9
**Total number of drip stains developed**				**162 (81+81)**

**Table 4:** Environmental conditions maintained within the laboratory for all the experiments undertaken.

**Atmospheric conditions**	
**Laboratory setting**	
Temperature	37 °C
Humidity	60%
Wind condition	Not windy

**Environmental condition (outside the laboratory)**	
Dry temperature	23 °C (approx.)
Wet temperature	26° C (approx.)
Relative humidity	77–78% (approx.)
Wind condition	Not windy

**Table 5:** Feature specification for the paper target surface used in the experiments (courtesy: JK Copier Paper Manufacturing).

**Feature**	**Measurement unit**	**Measurement value**
Actual substance	GSM	75.9
Thickness	μ	105
Bulk	cc/gm	1.38
Density	Gm/cc	0.72
Porosity	MI/Min	800
Size of each sheet	cm^2^	29.7×42
Weight of each sheet	kg	0.00936
Sheet color	sRGB Color model	White

**Tablee 6:** Intra variability in porcine blood that was used in the experiments.

**Porcine blood**	**Before the experiment [Blood at 37 °C and 60% humidity]**
Age since collection from a pig	1-day-old
PCV	0.39±0.01
Density, ×10^3^ kg/m^3^	1.069±0.022
Surface tension, mN/m	62.47±0.71
Whole blood viscosity, mN s/m^2^	4.001±0.008
Plasma viscosity, mN s/m^2^	1.482±0.003

**Table 7:** The mean and standard deviation of the length, breadth and total number of spines for 9 stain patterns formed by ejection of blood drop from a 2.5 cc subcutaneous syringe without needle filled to capacity with blood (250 ml) mixed with 2 mg of Warfarin at an impact angle of 60° and fall height of 40 cm.

**Stain parameters**	**Mean**	**Standard deviation**
Length	1.2556	.0527
Breadth	1.1222	.0441
Total Number of spines	17.4444	1.2360
