# Supplementary material for: Initial data release of regular blood drip stain created by varying fall height, angle of impact and source dimension
Source: Data Brief. 2016 Jul 6;8:1194–205. doi: 10.1016/j.dib.2016.07.003 (PMC4979045; doi:10.1016/j.dib.2016.07.003)
Supplement: Supplementary file 1 — Supplementary material [file mmc1.doc]

**CONFLICT OF INTEREST FORM**

**There was no conflict of interest.**
