# Supplementary material for: Initial data release of regular blood drip stain created by varying fall height, angle of impact and source dimension
Source: Data Brief. 2016 Jul 6;8:1194–205. doi: 10.1016/j.dib.2016.07.003 (PMC4979045; doi:10.1016/j.dib.2016.07.003)
Supplement: Supplementary file 2 — Supplementary material [file mmc2.zip › Bloodstain_dataset/Dataset Specifications.docx]

**Dataset Specification**

- Images of individual stains developed at different impact angles ( 30^o^, 60^o^, 90^o^) for each particular fall height (20 , 40 and 60 cms) have been uploaded in ‘DSCN*.jpeg ‘ format
- Figure 1 provides the structure used for the data arrangement/grouping

**Folder:** Bloodstain_dataset

**Folder:** Fresh

**Folder:** Heparin

**Folder:** Warfarin

**Folder:** Subcutaneous

**Folder:** Syringe with Needle

**Folder:** Syringe without Needle

**Figure 1**: The folder hierarchical structure developed for image archival

- ‘Dataset_Map.xlsx’ contains the image specifications, such as length, breadth, dosage, intended actual angle of impact, calculated angle of impact
- Folders named ‘Fresh’, ‘Heparin’ and ‘Warfarin’, consist of images of stains formed when a 2.5 cc. subcutaneous syringe with needle was allowed to eject a single drop of fresh pig blood, blood mixed with Heparin and blood mixed with Warfarin respectively. Folder ‘Warfarin’ and ‘Heparin’ consist of 45 datapoints/images each, while folder ‘Fresh’ consists of 18 datapoints. Table 1 reports the folder-wise break-up of the datapoints.

**Table 1:** A tabular listing of the content of each folder and subfolder used for data archival

| **Folder Name** | **Contents** |
| --- | --- |
| Fresh | \|  \| Fall Height \| \| \| \| --- \| --- \| --- \| --- \| \| Impact Angle -30^o^ \| 20 cm \| 40 cm \| 60 cm \|   Therefore, for impact angle 30^o^ – 3 images/stains/datapoints at different fall heights were created. Similar procedure was repeated for 60^o^ and 90^o^ impact angle. Total number of points – 3+3+3 = 9 datapoints/images/stains  A repeat set of 9 created = 9 x 2= 18 ; Total = 18 JPEG images [ All stains were created with a Subcutaneous Syringe with needle ] |
| Heparin | Each impact angle combination with each of three fall heights result in total 9 images. Five different dosage of Heparin were applied to blood and 9 samples were created with each of 5 processed blood set. Total 9 x 5 = 45 samples were thus created.[ All stains were created with a Subcutaneous Syringe with needle ] |
| Warfarin | Each impact angle combination with each of three fall heights result in total 9 images. Five different dosage of Warfarin were applied to blood and 9 samples were created with each of 5 processed blood set. Total 9 x 5 = 45 samples were thus created. [ All stains were created with a Subcutaneous Syringe with needle ] |
| Subcutaneous | \| **Folder :** Syringe with Needle \| 9 images of blood drops from a 2.5 cc. Subcutaneous Syringe **with** Needle were created with blood processed with Warfarin (2mg. of Warfarin in 250 ml. blood) \| \| --- \| --- \| \| **Folder** **:** Syringe without Needle \| 9 images of blood drops from a 2.5 cc. Subcutaneous Syringe **without** Needle were created with blood processed with Warfarin (2mg. of Warfarin in 250 ml. blood) \| |
| ***** All experiments were performed in controlled environmental conditions. All images are in JPEG format. Size of Each Image- 500 x 760 pixels and 300 dpi resolution  ** All drop stains were developed on the same visibly non-porous, smooth target surface. | |

- The ‘Dataset_map.xlsx’ Sheet 1 consists of the details of the stain patterns present in Folders, ‘Fresh’, ‘Heparin’ and ‘Warfarin’. The details of each stain pattern consist of the length of the stain, the breadth of the stain, dosage, total number of satellites, intended actual angle of impact, and calculated angle of impact. In the dosage column, ‘Pure’ represents fresh pig blood. The significance of other dosage abbreviations with respect to Heparin and Warfarin are summarized in Table 2.
- Folder ‘Subcutaneous’ consists of two subfolders. They are, ‘Syringe with Needle’ and ‘Syringe without Needle’. Each folder, ‘Syringe with Needle’ and ‘Syringe without Needle’, consist of 9 images each. Table 1 displays the folder-wise break-up of the images.

| **Warfarin** | **Heparin** |
| --- | --- |
| \| **Abbreviation** \| **Meaning** \| \| --- \| --- \| \| Pure + 2 \| 2mg. of powdered Warfarin was mixed with 250ml of fresh pig blood \| \| Pure + 4 \| 4mg. of powdered Warfarin was mixed with 250ml of fresh pig blood \| \| Pure + 6 \| 6mg. of powdered Warfarin was mixed with 250ml of fresh pig blood \| \| Pure + 8 \| 8mg. of powdered Warfarin was mixed with 250ml of fresh pig blood \| \| Pure + 10 \| 10mg. of powdered Warfarin was mixed with 250ml of fresh pig blood \| | \| **Abbreviation** \| **Meaning** \| \| --- \| --- \| \| Pure + 2 \| 260 I.U. of Heparin was mixed with 250ml of fresh pig blood \| \| Pure + 4 \| 520 I.U. of Heparin was mixed with 250 ml of fresh pig blood \| \| Pure + 6 \| 780 I.U. of Heparin was mixed with 250 ml of fresh pig blood \| \| Pure + 8 \| 1040 I.U. of Heparin was mixed with 250 ml of fresh pig blood \| \| Pure + 10 \| 1300 I.U. of Heparin was mixed with 250 ml of fresh pig blood \| |
| * 130 I.U.(International Units) = 1 mg[1] | |

**Table 2:** Chemical specific explanation of abbreviations used in the Dataset_map.xlsx file

**N.B**.: For details relating to calculation of the length, breadth, angle of impact (calculated, intended), total number of satellites please refer to the Data Descriptor titled ‘Initial data release to analyze effect of Angle, Fall Height & Source Dimension on shape of regular blood drip stain’.

**TO OBTAIN THE ENTIRE DATASET PLEASE FEEL FREE TO CONTACT US at** [**1954samir@gmail.com**](mailto:1954samir@gmail.com)

**Reference**

1. The Indian Pharmacopoeia Commission, Government of India, Ministry of Health and Family Welfare, INDIAN PHARMACOPOEIA 2007, 2:567, Retrieved from <http://ajprd.com/downloadebooks_pdf/9.pdf>, (2007) (18.09.2014)
