# Supplementary figures and images for: Initial data release of regular blood drip stain created by varying fall height, angle of impact and source dimension (part 2 of 2)
Source: Data Brief. 2016 Jul 6;8:1194–205. doi: 10.1016/j.dib.2016.07.003 (PMC4979045; doi:10.1016/j.dib.2016.07.003)

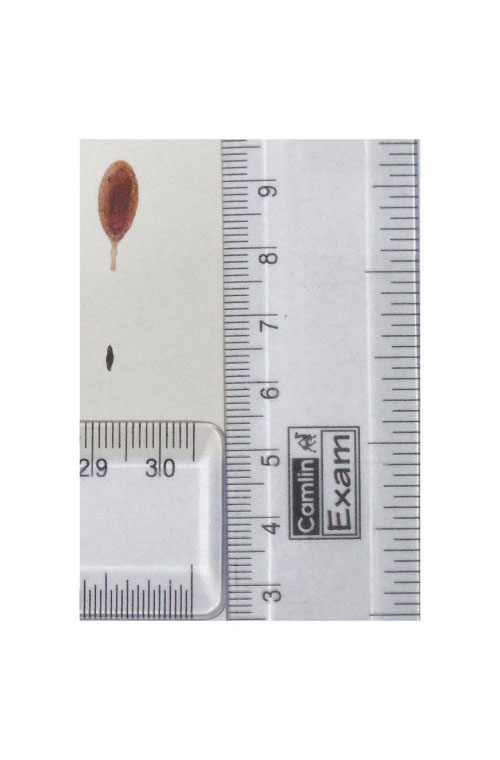

Supplement: Supplementary file 2 — Supplementary material [file mmc2.zip › Bloodstain_dataset/Warfarin/DSCN0874.jpg]

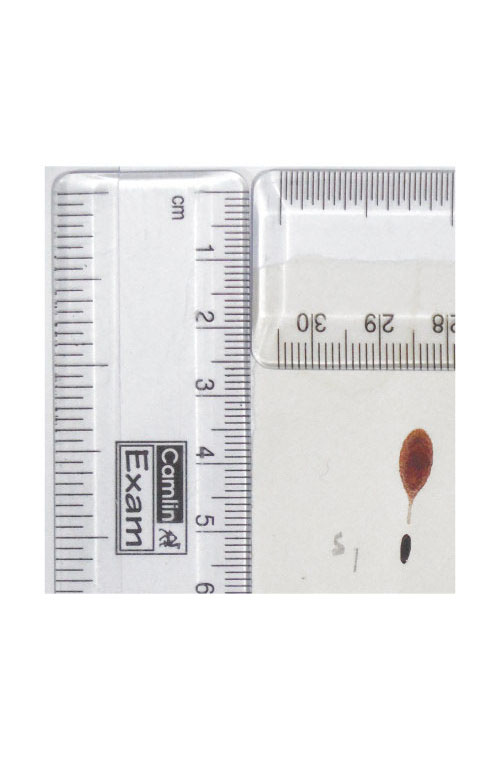

Supplement: Supplementary file 2 — Supplementary material [file mmc2.zip › Bloodstain_dataset/Warfarin/DSCN0884.jpg]

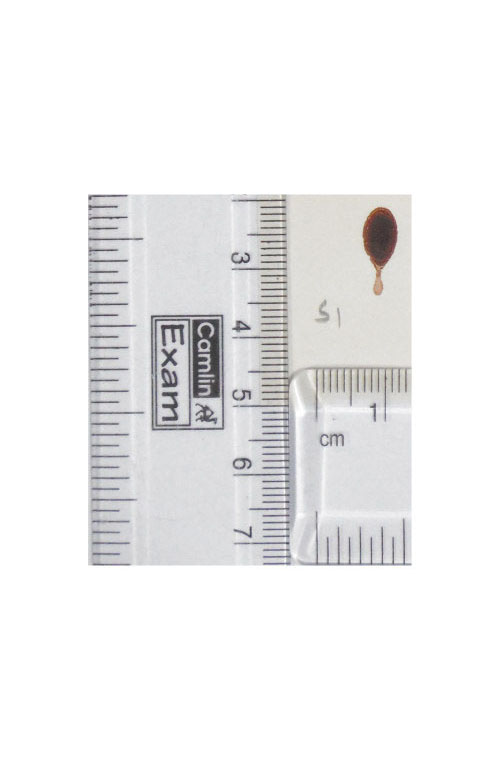

Supplement: Supplementary file 2 — Supplementary material [file mmc2.zip › Bloodstain_dataset/Warfarin/DSCN0888.jpg]

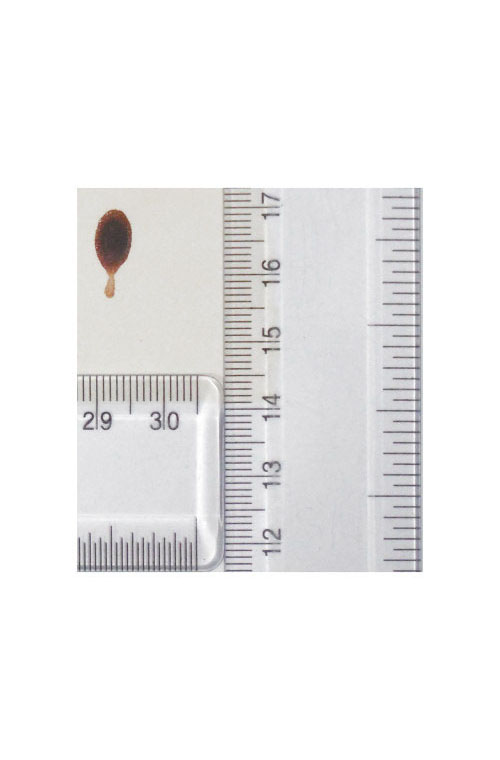

Supplement: Supplementary file 2 — Supplementary material [file mmc2.zip › Bloodstain_dataset/Warfarin/DSCN0891.jpg]

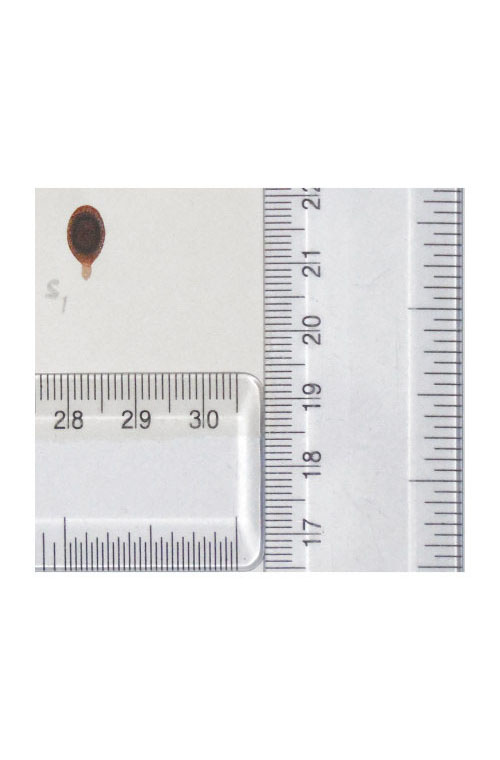

Supplement: Supplementary file 2 — Supplementary material [file mmc2.zip › Bloodstain_dataset/Warfarin/DSCN0896.jpg]

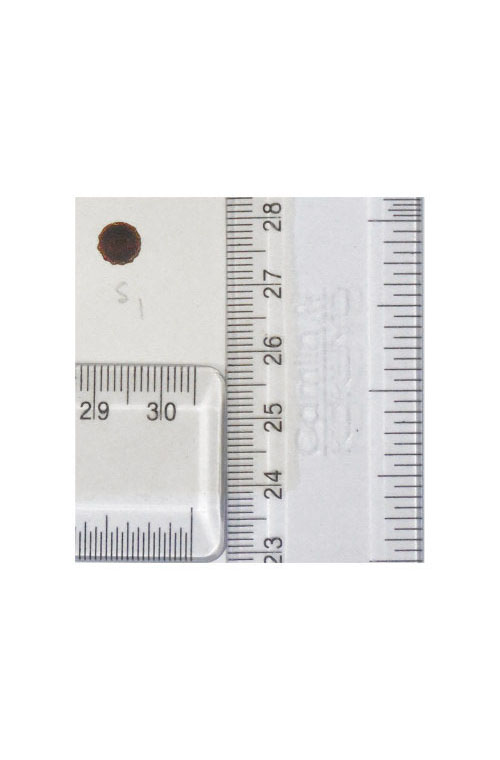

Supplement: Supplementary file 2 — Supplementary material [file mmc2.zip › Bloodstain_dataset/Warfarin/DSCN0936.jpg]

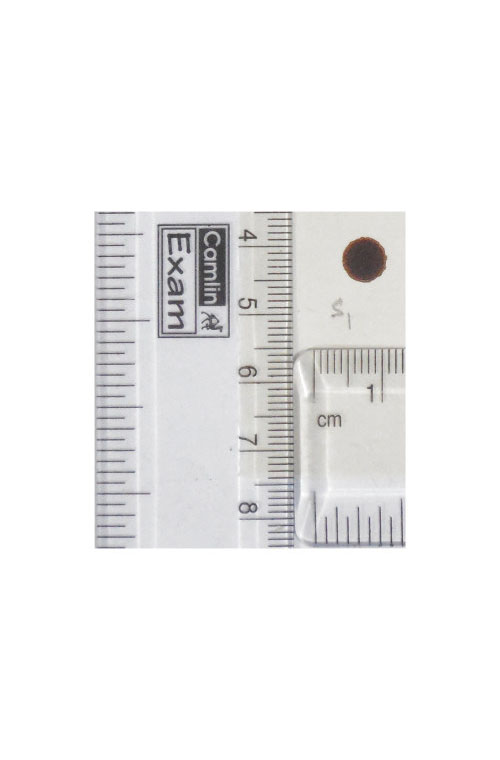

Supplement: Supplementary file 2 — Supplementary material [file mmc2.zip › Bloodstain_dataset/Warfarin/DSCN0942.jpg]

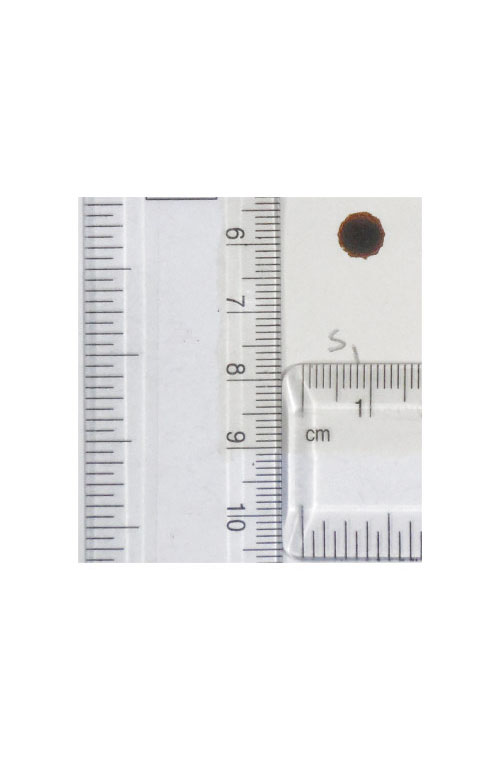

Supplement: Supplementary file 2 — Supplementary material [file mmc2.zip › Bloodstain_dataset/Warfarin/DSCN0945.jpg]

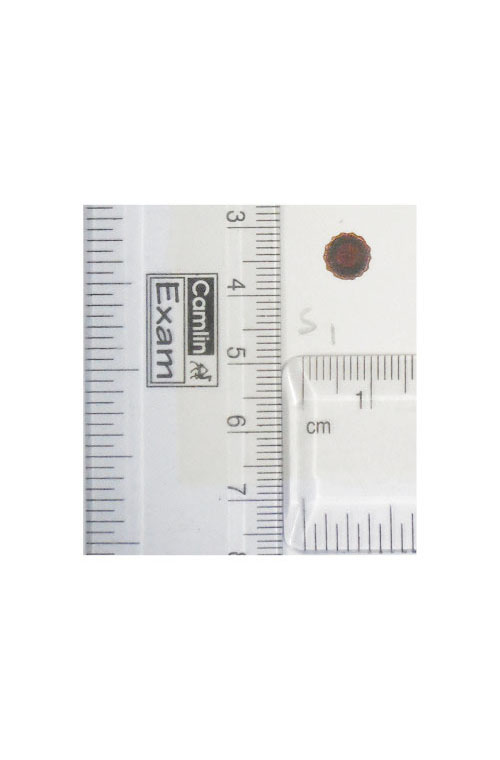

Supplement: Supplementary file 2 — Supplementary material [file mmc2.zip › Bloodstain_dataset/Warfarin/DSCN0950.jpg]

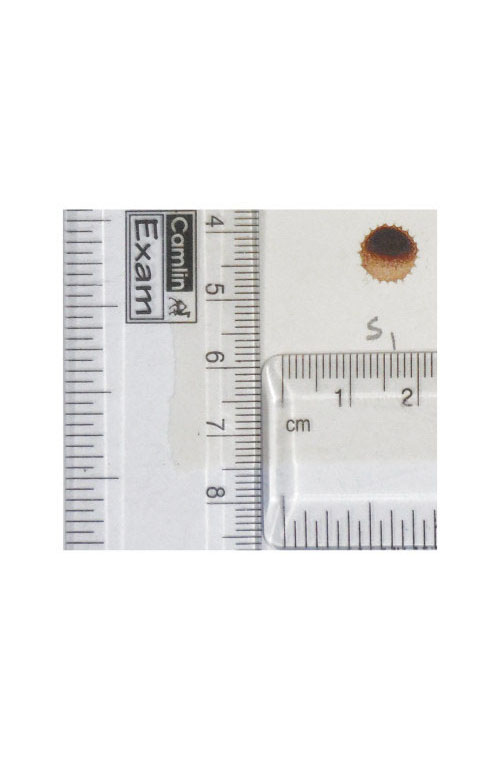

Supplement: Supplementary file 2 — Supplementary material [file mmc2.zip › Bloodstain_dataset/Warfarin/DSCN0961.jpg]

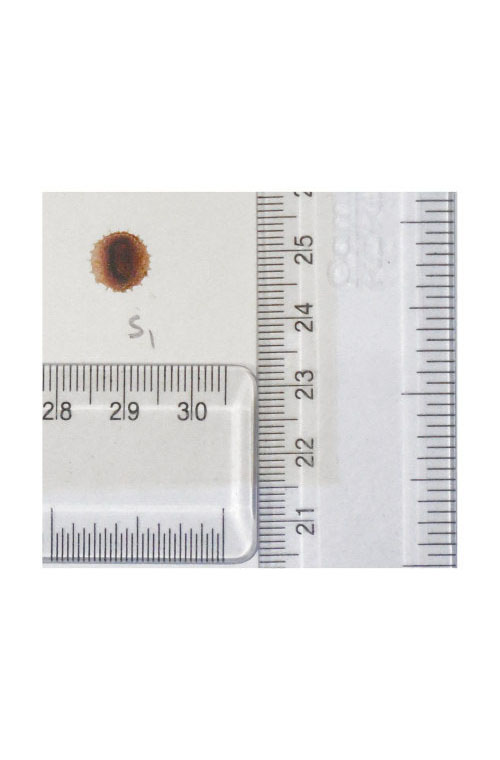

Supplement: Supplementary file 2 — Supplementary material [file mmc2.zip › Bloodstain_dataset/Warfarin/DSCN0966.jpg]

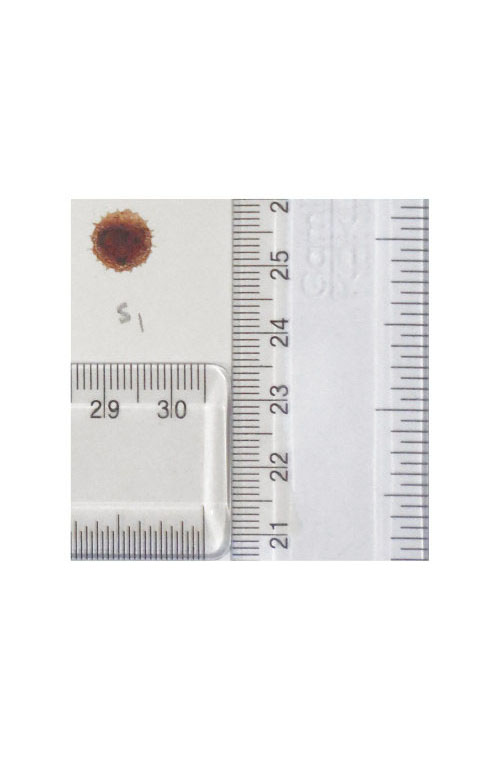

Supplement: Supplementary file 2 — Supplementary material [file mmc2.zip › Bloodstain_dataset/Warfarin/DSCN0969.jpg]

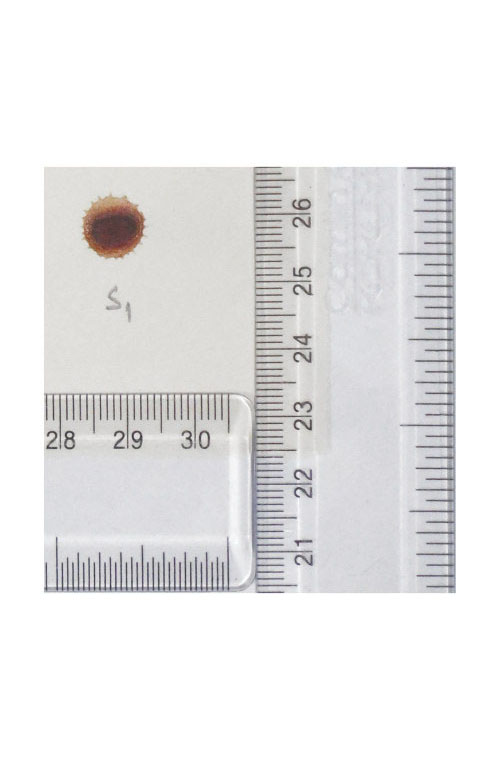

Supplement: Supplementary file 2 — Supplementary material [file mmc2.zip › Bloodstain_dataset/Warfarin/DSCN0972.jpg]

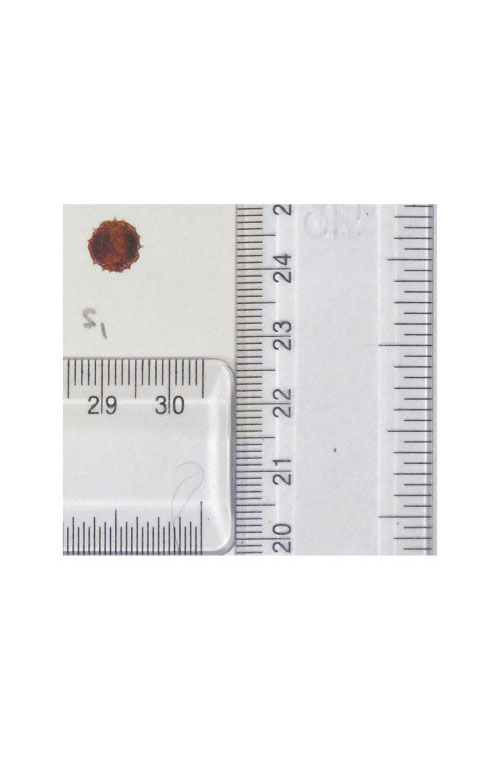

Supplement: Supplementary file 2 — Supplementary material [file mmc2.zip › Bloodstain_dataset/Warfarin/DSCN0983.jpg]

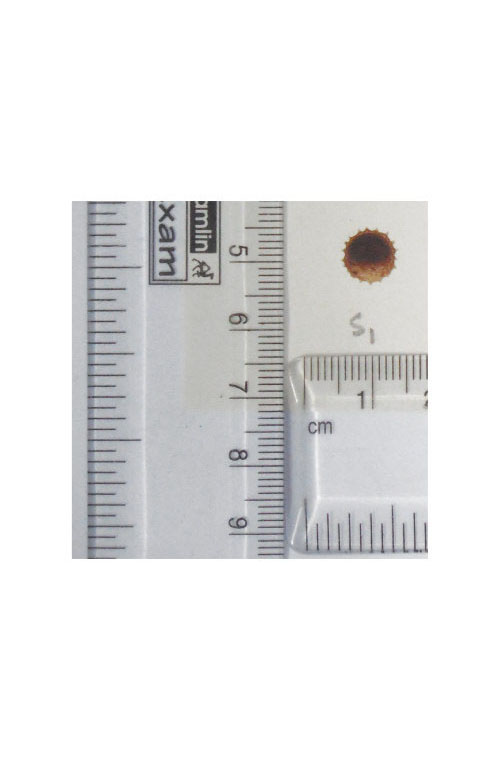

Supplement: Supplementary file 2 — Supplementary material [file mmc2.zip › Bloodstain_dataset/Warfarin/DSCN0987.jpg]

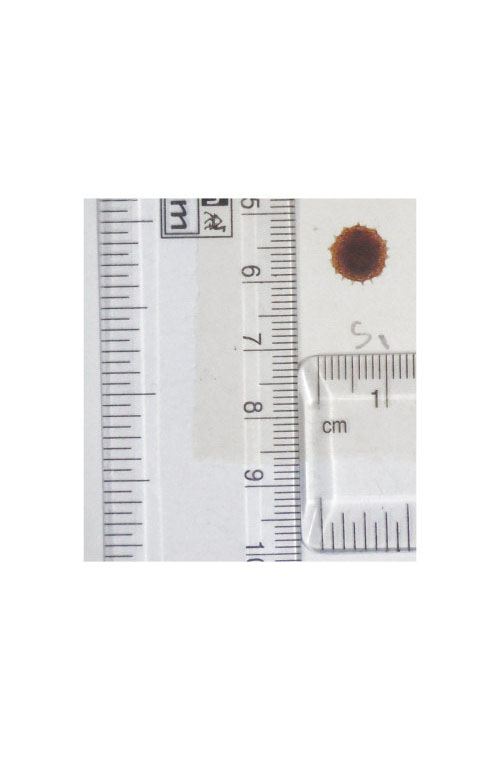

Supplement: Supplementary file 2 — Supplementary material [file mmc2.zip › Bloodstain_dataset/Warfarin/DSCN0991.jpg]

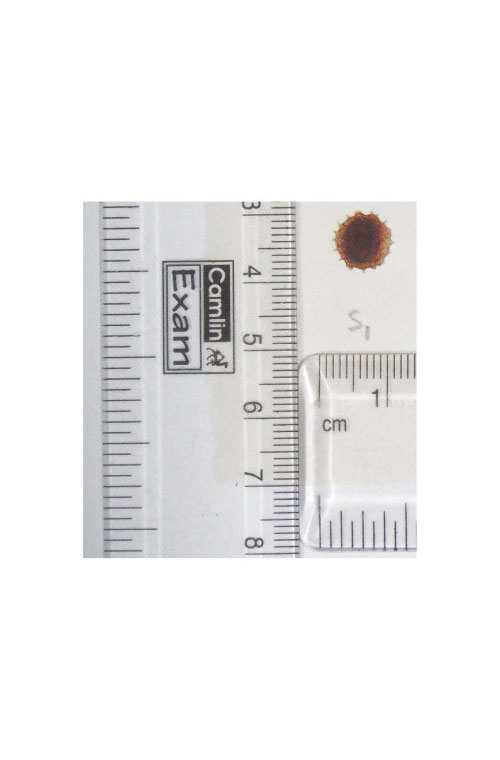

Supplement: Supplementary file 2 — Supplementary material [file mmc2.zip › Bloodstain_dataset/Warfarin/DSCN0993.jpg]
