# Supplementary figures and images for: Initial data release of regular blood drip stain created by varying fall height, angle of impact and source dimension (part 1 of 2)
Source: Data Brief. 2016 Jul 6;8:1194–205. doi: 10.1016/j.dib.2016.07.003 (PMC4979045; doi:10.1016/j.dib.2016.07.003)

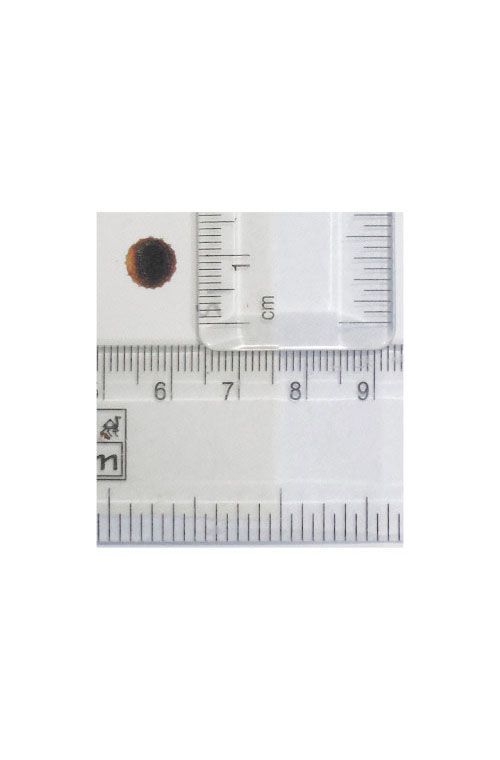

Supplement: Supplementary file 2 — Supplementary material [file mmc2.zip › Bloodstain_dataset/Fresh/DSCN0723.jpg]

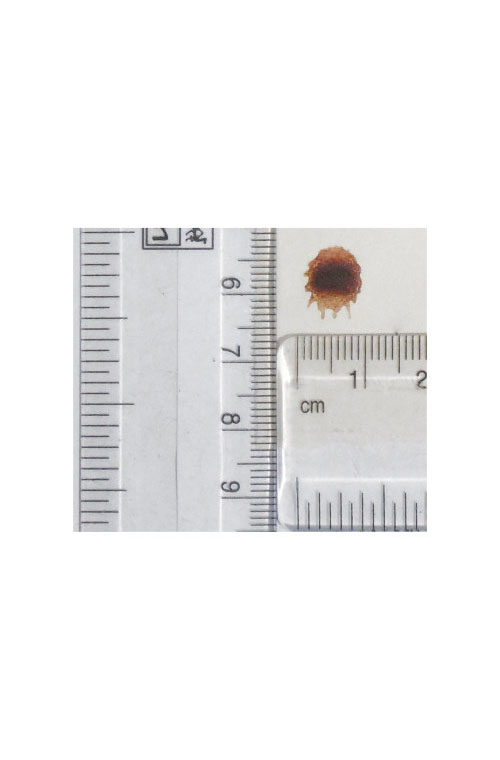

Supplement: Supplementary file 2 — Supplementary material [file mmc2.zip › Bloodstain_dataset/Fresh/DSCN0761.jpg]

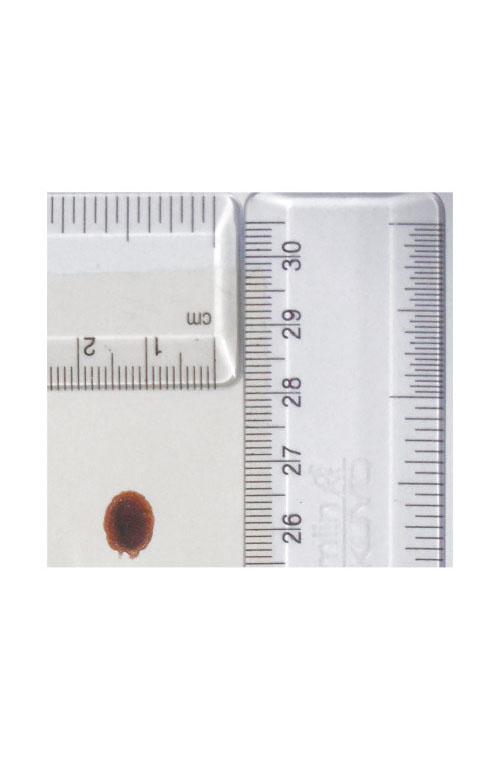

Supplement: Supplementary file 2 — Supplementary material [file mmc2.zip › Bloodstain_dataset/Fresh/DSCN0789.jpg]

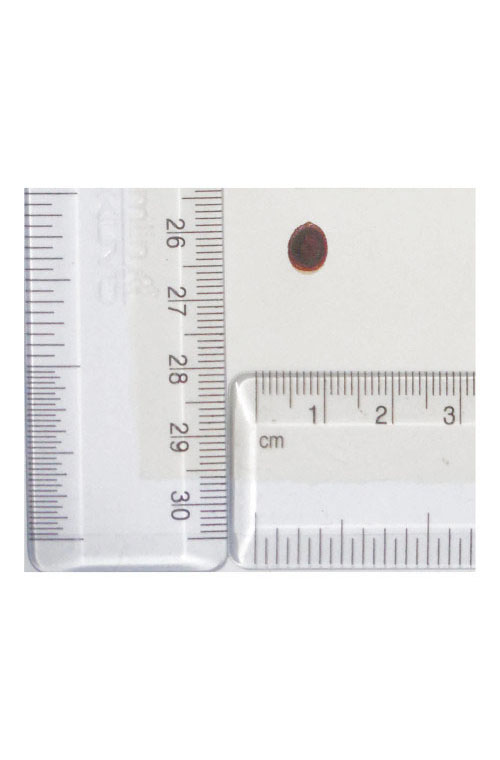

Supplement: Supplementary file 2 — Supplementary material [file mmc2.zip › Bloodstain_dataset/Fresh/DSCN0814.jpg]

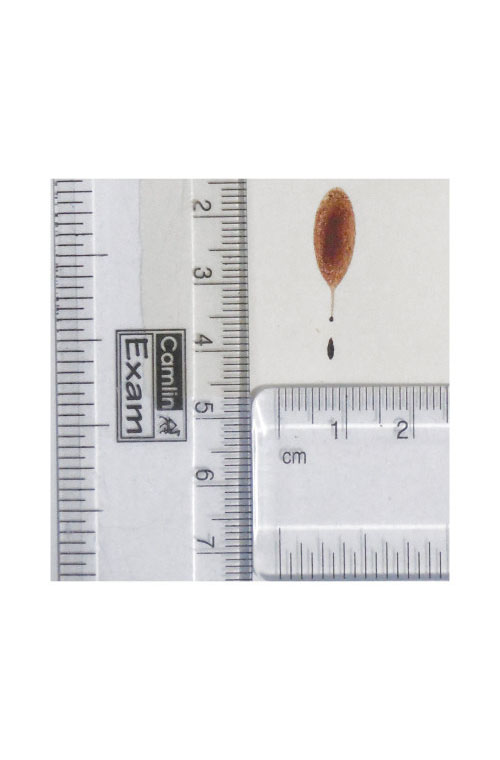

Supplement: Supplementary file 2 — Supplementary material [file mmc2.zip › Bloodstain_dataset/Fresh/DSCN0831.jpg]

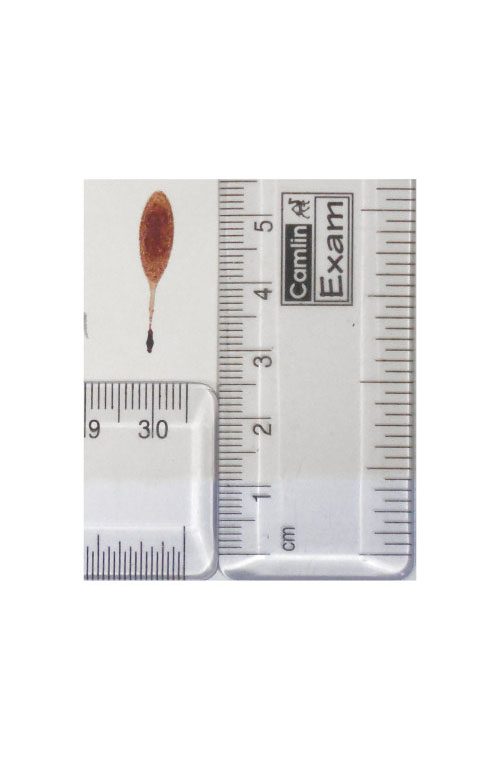

Supplement: Supplementary file 2 — Supplementary material [file mmc2.zip › Bloodstain_dataset/Fresh/DSCN0855.jpg]

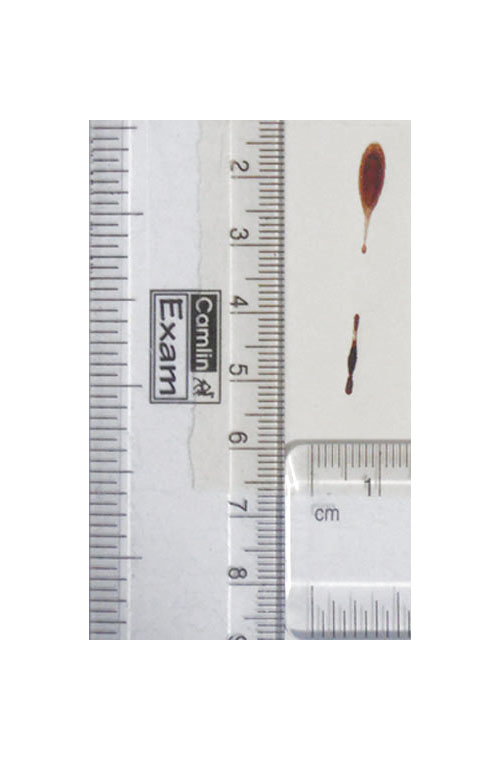

Supplement: Supplementary file 2 — Supplementary material [file mmc2.zip › Bloodstain_dataset/Fresh/DSCN0877.jpg]

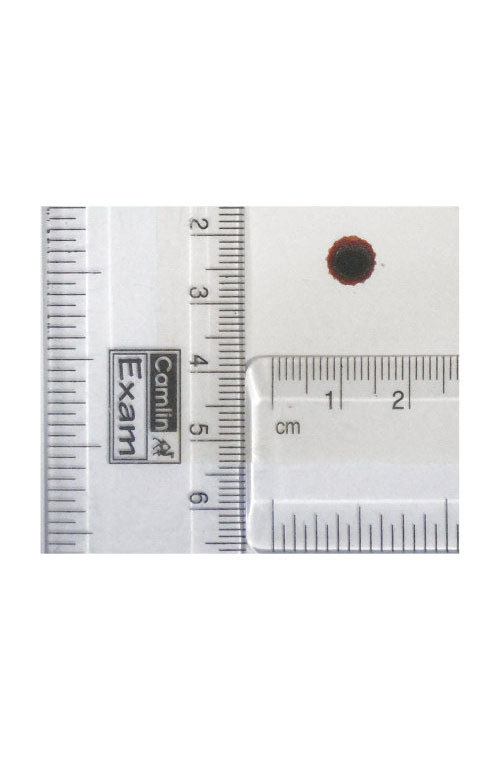

Supplement: Supplementary file 2 — Supplementary material [file mmc2.zip › Bloodstain_dataset/Fresh/DSCN0900.jpg]

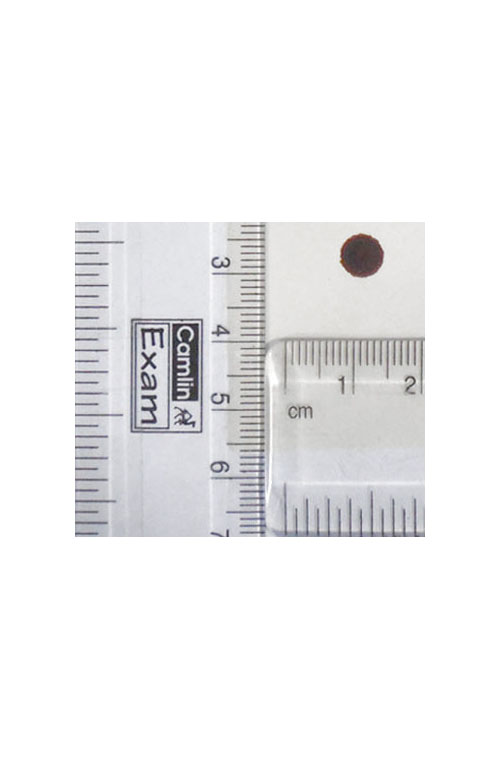

Supplement: Supplementary file 2 — Supplementary material [file mmc2.zip › Bloodstain_dataset/Fresh/DSCN0926.jpg]

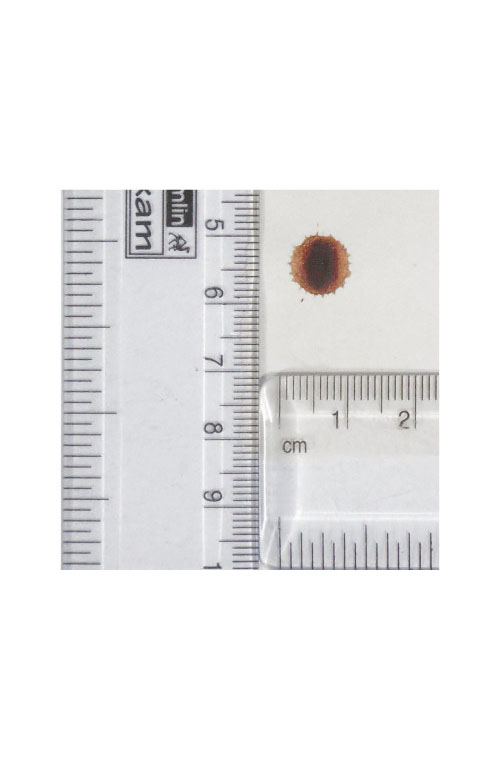

Supplement: Supplementary file 2 — Supplementary material [file mmc2.zip › Bloodstain_dataset/Fresh/DSCN0953.jpg]

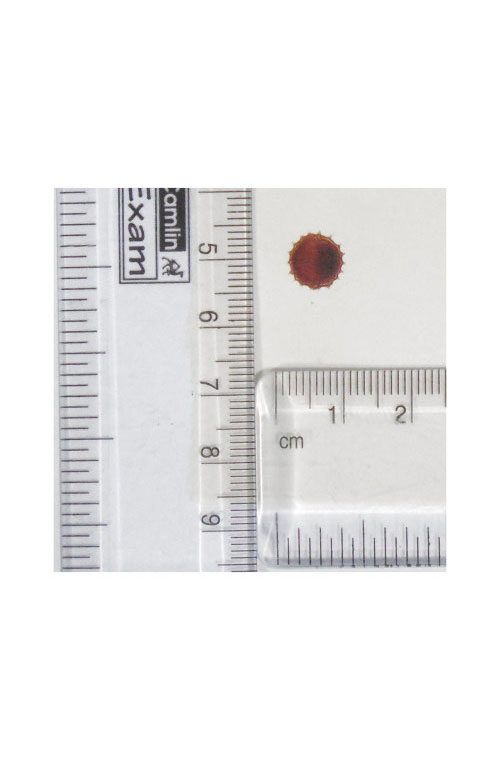

Supplement: Supplementary file 2 — Supplementary material [file mmc2.zip › Bloodstain_dataset/Fresh/DSCN0975.jpg]

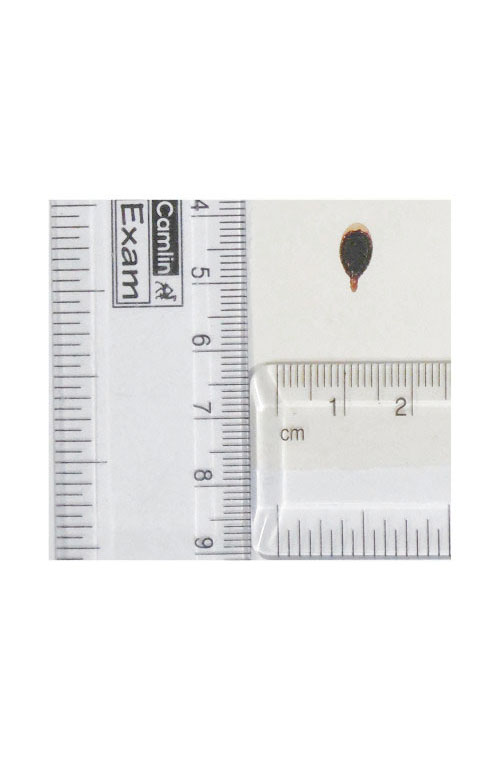

Supplement: Supplementary file 2 — Supplementary material [file mmc2.zip › Bloodstain_dataset/Fresh/DSCN0998.jpg]

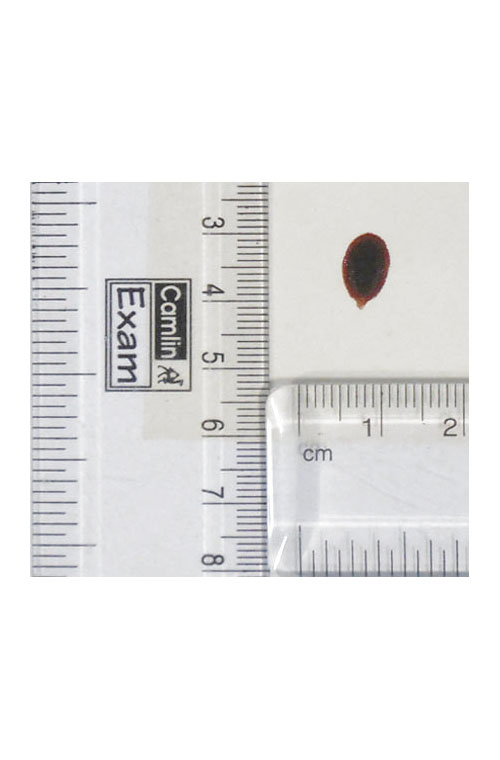

Supplement: Supplementary file 2 — Supplementary material [file mmc2.zip › Bloodstain_dataset/Fresh/DSCN1014.jpg]

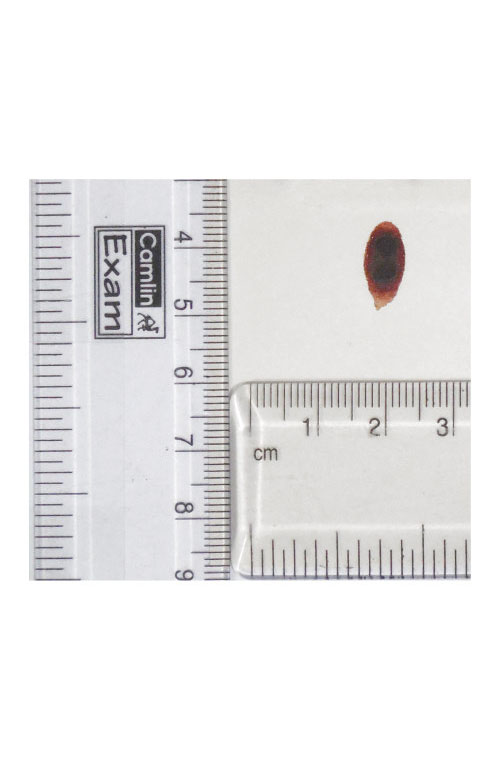

Supplement: Supplementary file 2 — Supplementary material [file mmc2.zip › Bloodstain_dataset/Fresh/DSCN1036.jpg]

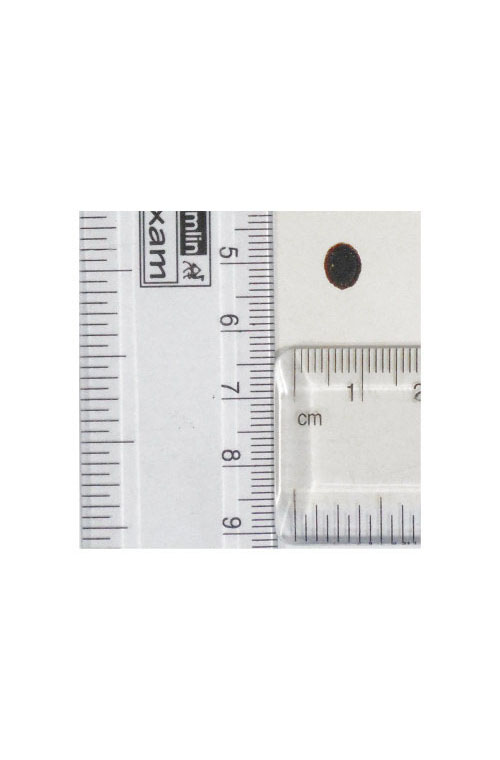

Supplement: Supplementary file 2 — Supplementary material [file mmc2.zip › Bloodstain_dataset/Fresh/DSCN1058.jpg]

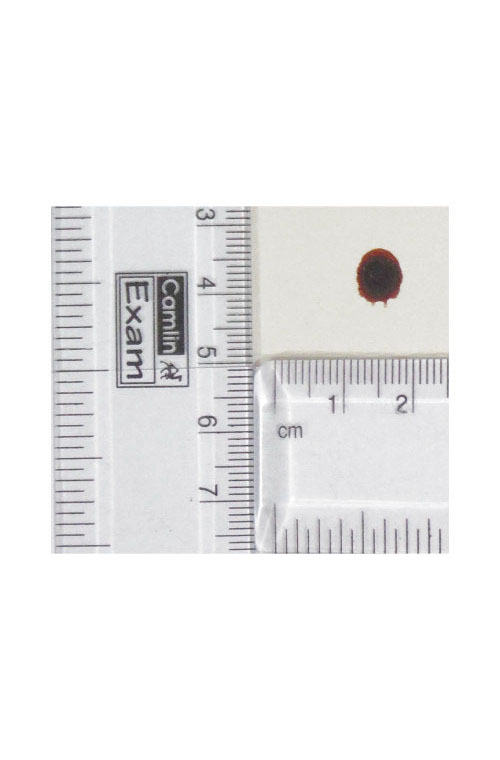

Supplement: Supplementary file 2 — Supplementary material [file mmc2.zip › Bloodstain_dataset/Fresh/DSCN1081.jpg]

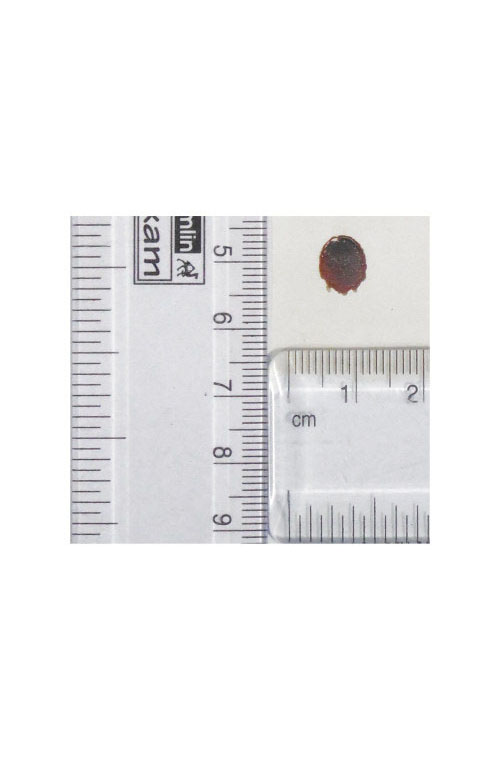

Supplement: Supplementary file 2 — Supplementary material [file mmc2.zip › Bloodstain_dataset/Fresh/DSCN1100.jpg]

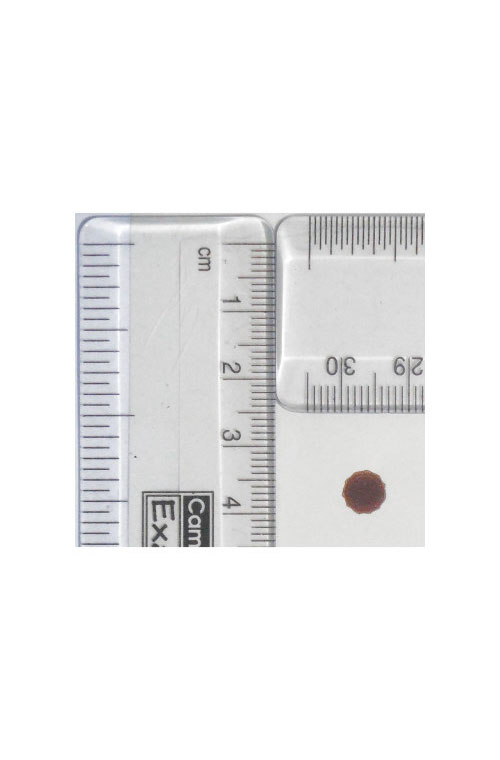

Supplement: Supplementary file 2 — Supplementary material [file mmc2.zip › Bloodstain_dataset/Fresh/DSCN1157.jpg]

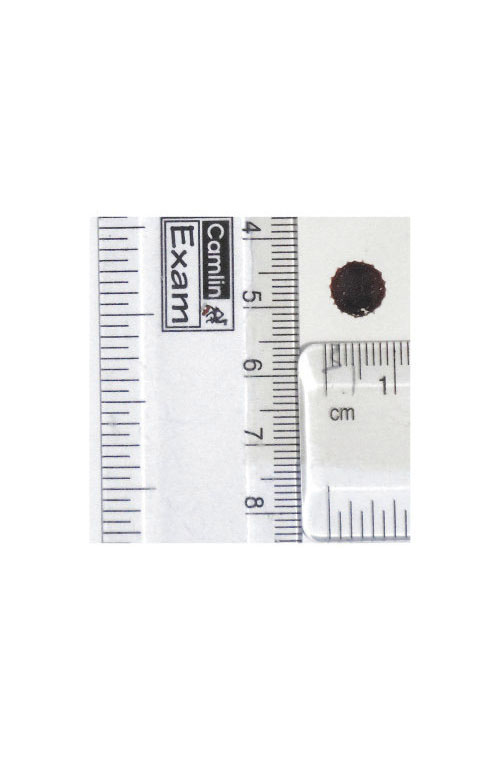

Supplement: Supplementary file 2 — Supplementary material [file mmc2.zip › Bloodstain_dataset/Heparin/DSCN0724.jpg]

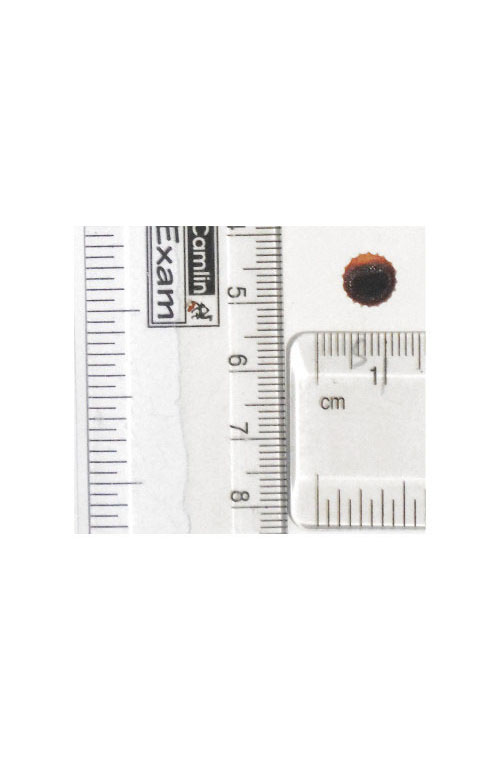

Supplement: Supplementary file 2 — Supplementary material [file mmc2.zip › Bloodstain_dataset/Heparin/DSCN0728.jpg]

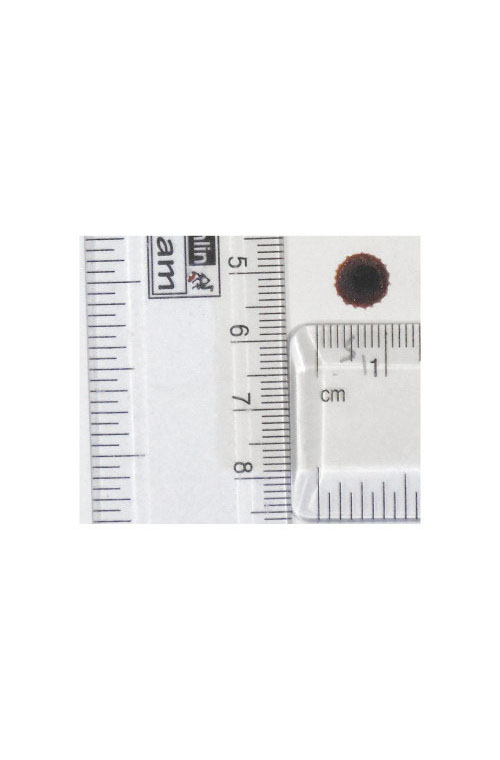

Supplement: Supplementary file 2 — Supplementary material [file mmc2.zip › Bloodstain_dataset/Heparin/DSCN0730.jpg]

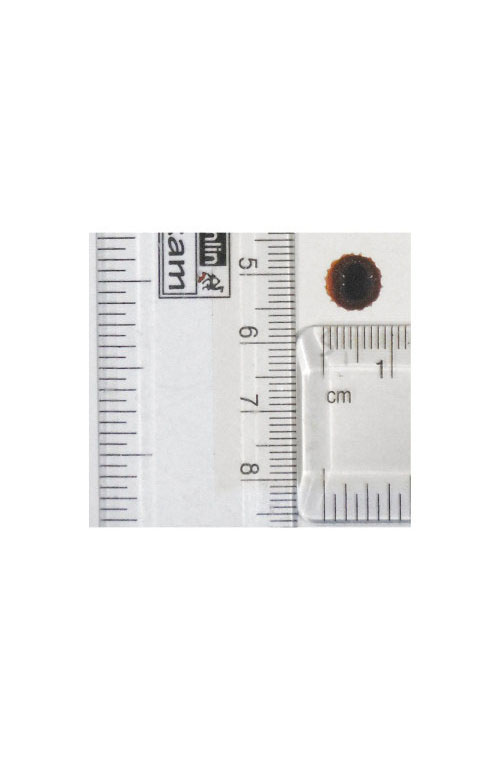

Supplement: Supplementary file 2 — Supplementary material [file mmc2.zip › Bloodstain_dataset/Heparin/DSCN0731.jpg]

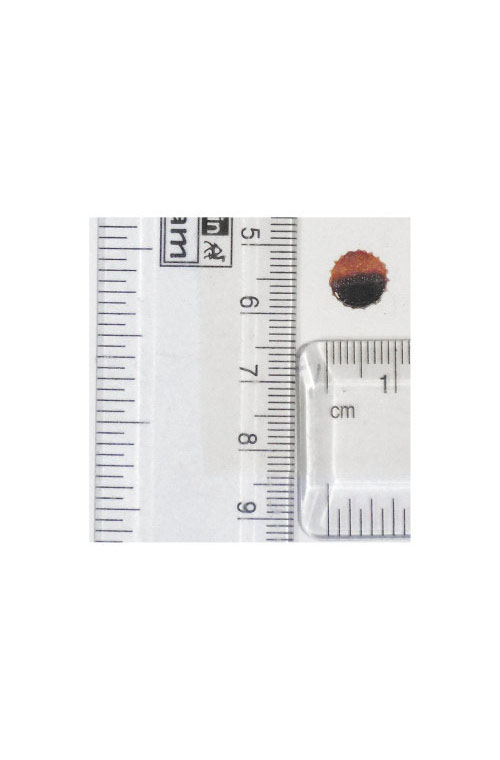

Supplement: Supplementary file 2 — Supplementary material [file mmc2.zip › Bloodstain_dataset/Heparin/DSCN0733.jpg]

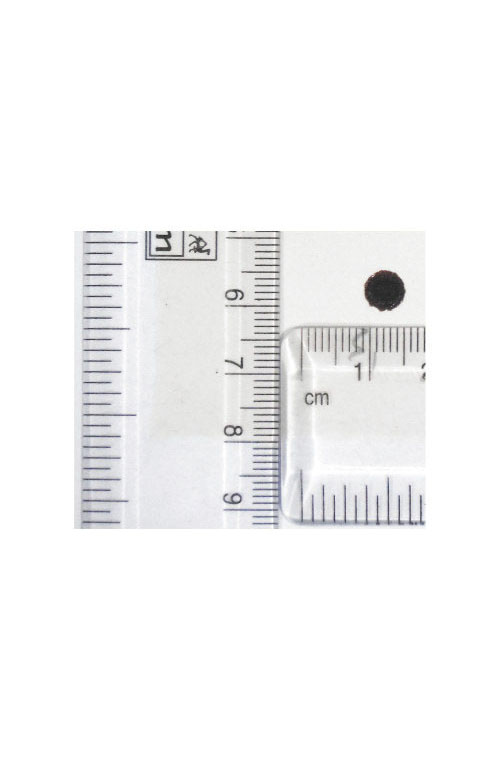

Supplement: Supplementary file 2 — Supplementary material [file mmc2.zip › Bloodstain_dataset/Heparin/DSCN0746.jpg]

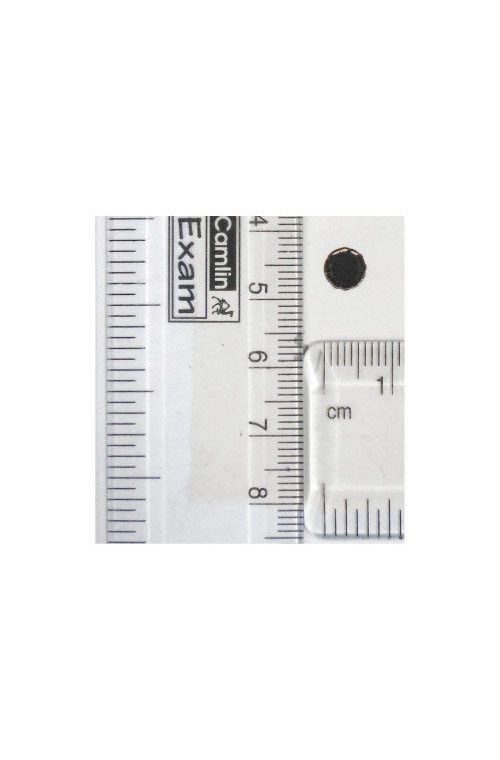

Supplement: Supplementary file 2 — Supplementary material [file mmc2.zip › Bloodstain_dataset/Heparin/DSCN0749.jpg]

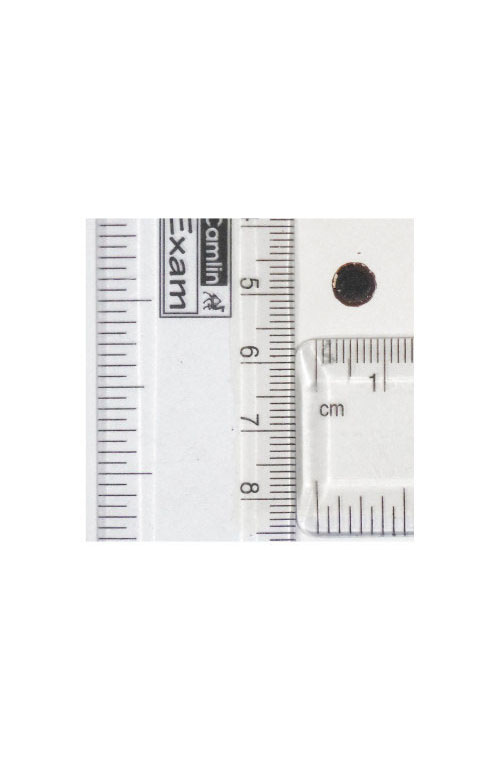

Supplement: Supplementary file 2 — Supplementary material [file mmc2.zip › Bloodstain_dataset/Heparin/DSCN0752.jpg]

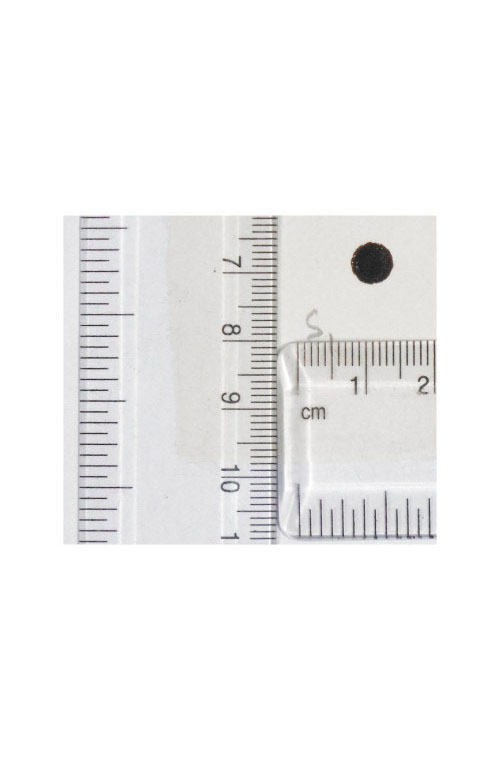

Supplement: Supplementary file 2 — Supplementary material [file mmc2.zip › Bloodstain_dataset/Heparin/DSCN0754.jpg]

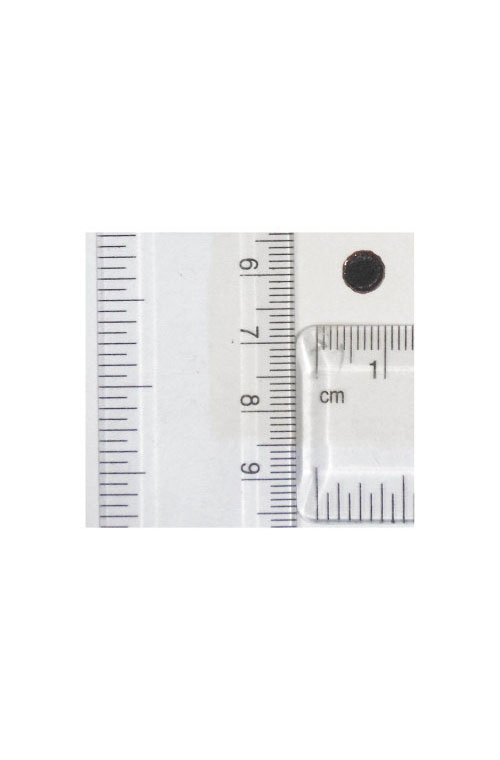

Supplement: Supplementary file 2 — Supplementary material [file mmc2.zip › Bloodstain_dataset/Heparin/DSCN0758.jpg]

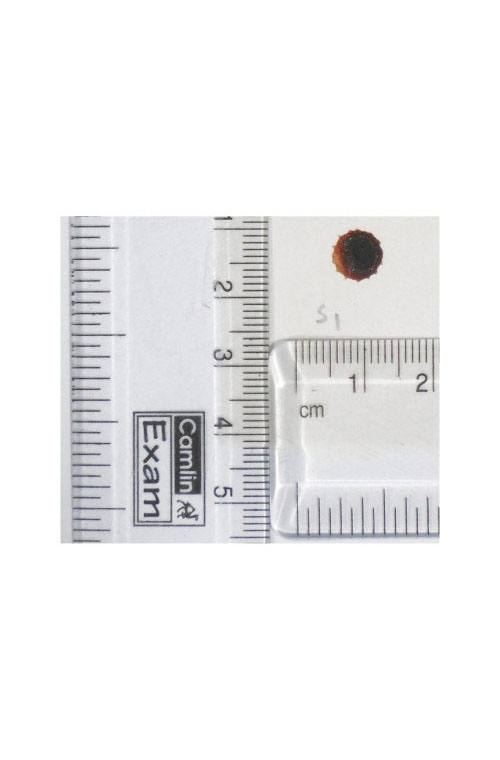

Supplement: Supplementary file 2 — Supplementary material [file mmc2.zip › Bloodstain_dataset/Heparin/DSCN0906.jpg]

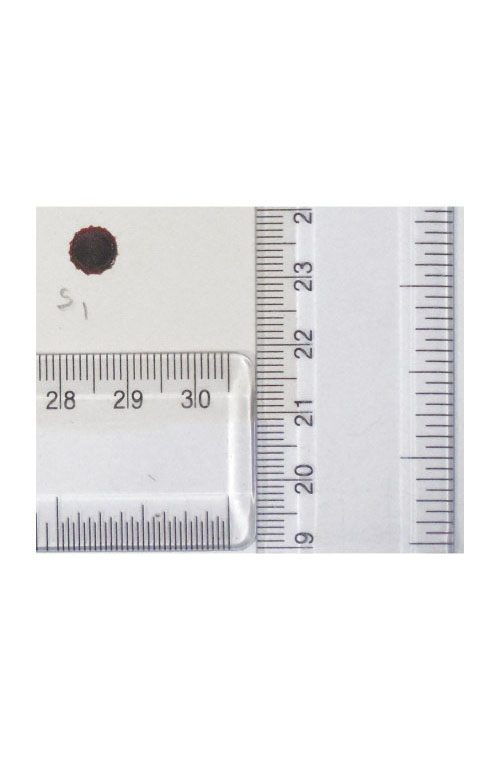

Supplement: Supplementary file 2 — Supplementary material [file mmc2.zip › Bloodstain_dataset/Heparin/DSCN0908.jpg]

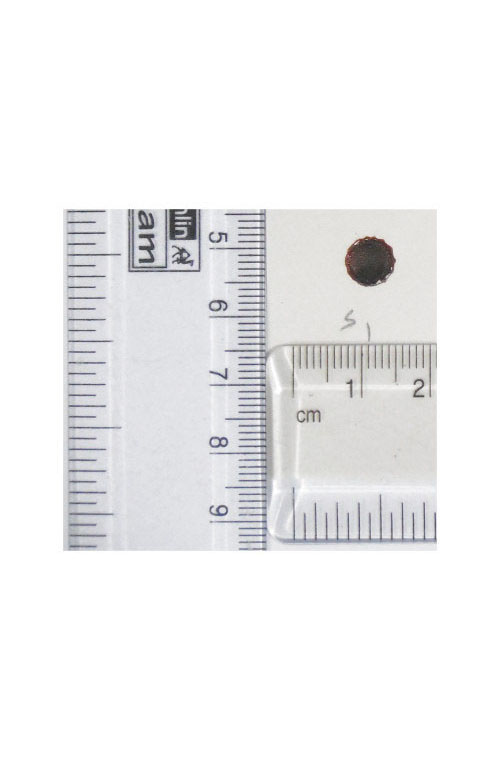

Supplement: Supplementary file 2 — Supplementary material [file mmc2.zip › Bloodstain_dataset/Heparin/DSCN0911.jpg]

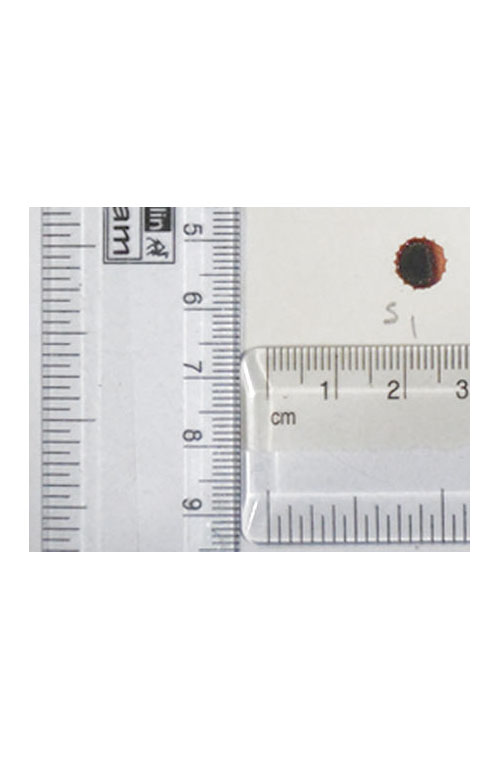

Supplement: Supplementary file 2 — Supplementary material [file mmc2.zip › Bloodstain_dataset/Heparin/DSCN0915.jpg]

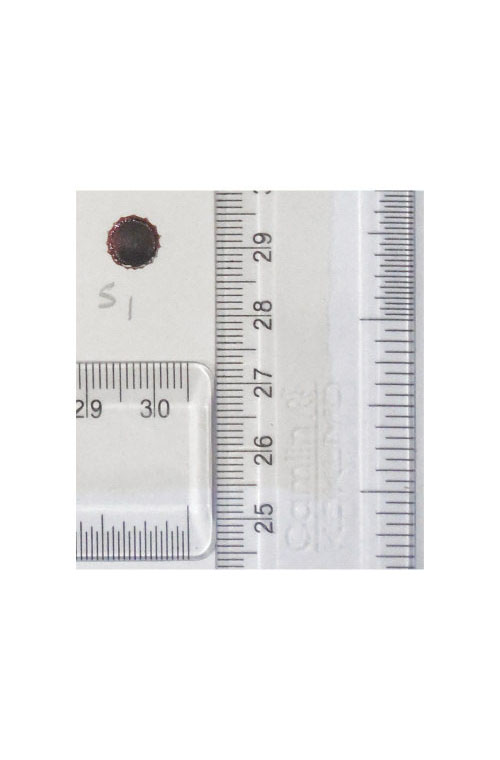

Supplement: Supplementary file 2 — Supplementary material [file mmc2.zip › Bloodstain_dataset/Heparin/DSCN0919.jpg]

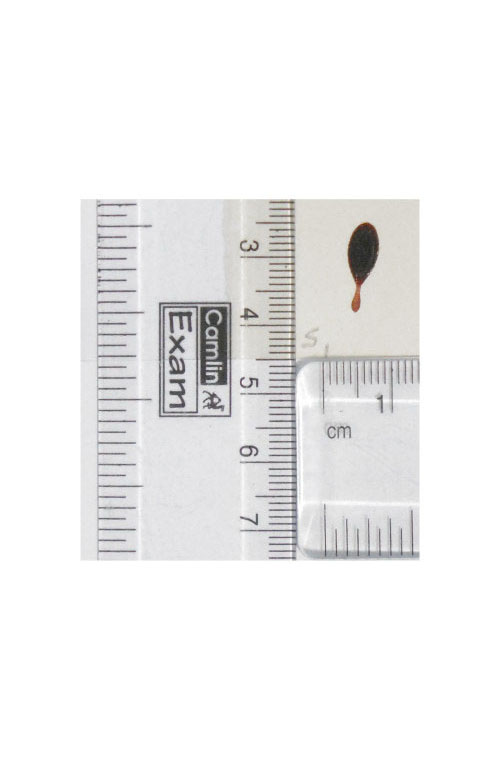

Supplement: Supplementary file 2 — Supplementary material [file mmc2.zip › Bloodstain_dataset/Heparin/DSCN1000.jpg]

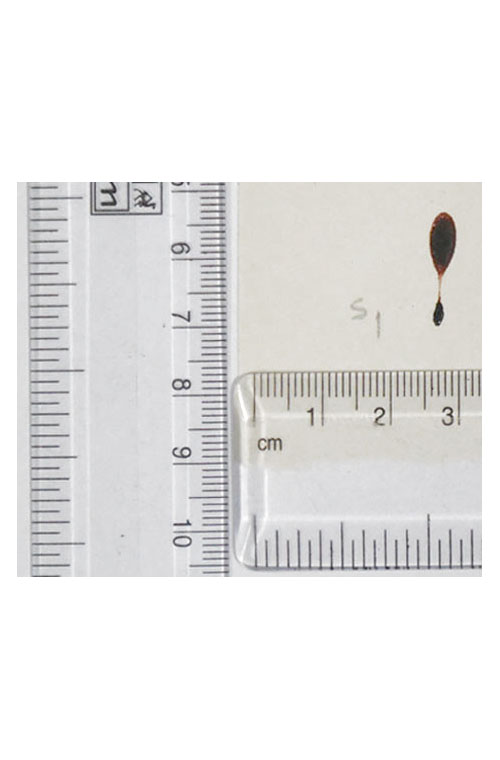

Supplement: Supplementary file 2 — Supplementary material [file mmc2.zip › Bloodstain_dataset/Heparin/DSCN1003.jpg]

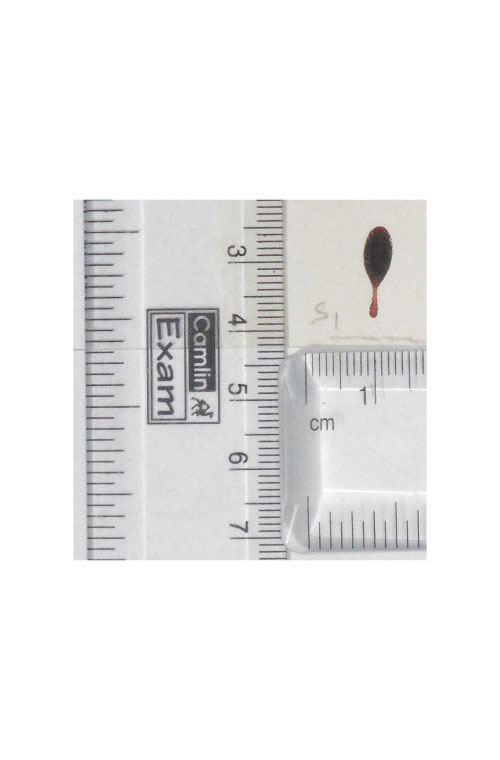

Supplement: Supplementary file 2 — Supplementary material [file mmc2.zip › Bloodstain_dataset/Heparin/DSCN1007.jpg]

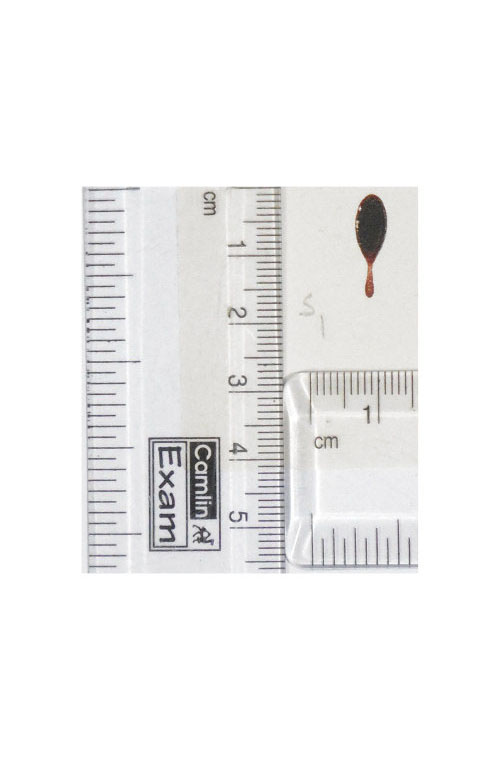

Supplement: Supplementary file 2 — Supplementary material [file mmc2.zip › Bloodstain_dataset/Heparin/DSCN1009.jpg]

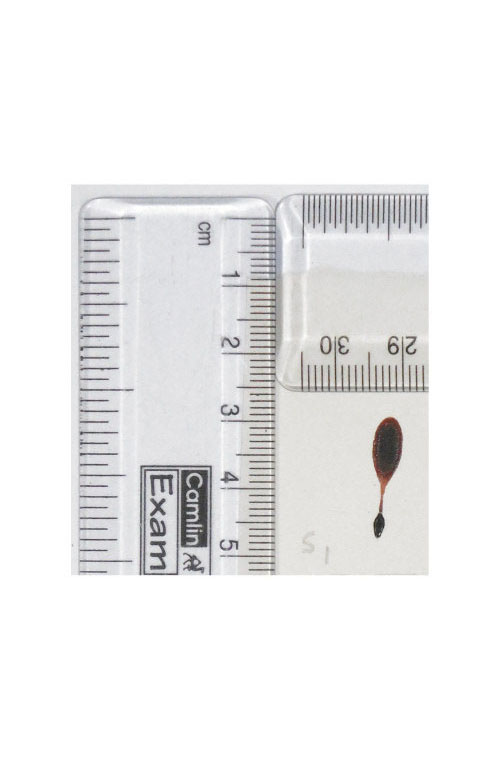

Supplement: Supplementary file 2 — Supplementary material [file mmc2.zip › Bloodstain_dataset/Heparin/DSCN1011.jpg]

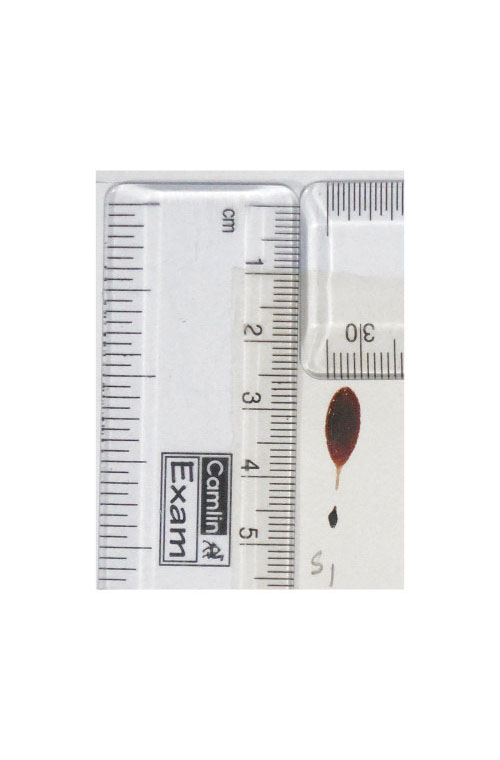

Supplement: Supplementary file 2 — Supplementary material [file mmc2.zip › Bloodstain_dataset/Heparin/DSCN1019.jpg]

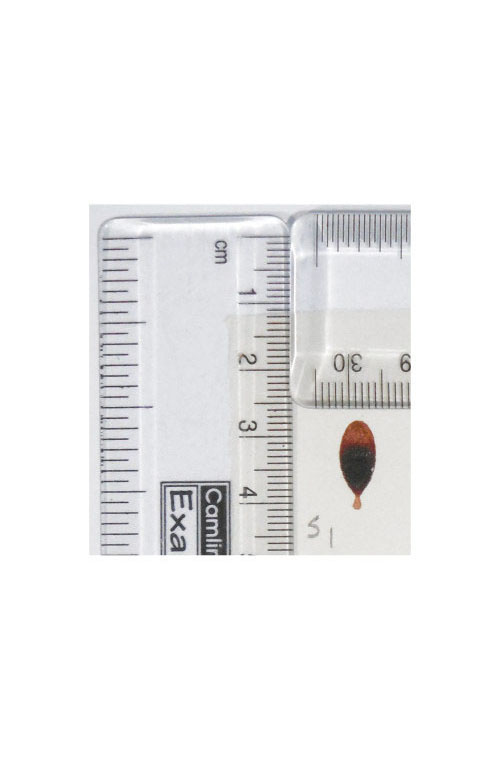

Supplement: Supplementary file 2 — Supplementary material [file mmc2.zip › Bloodstain_dataset/Heparin/DSCN1023.jpg]

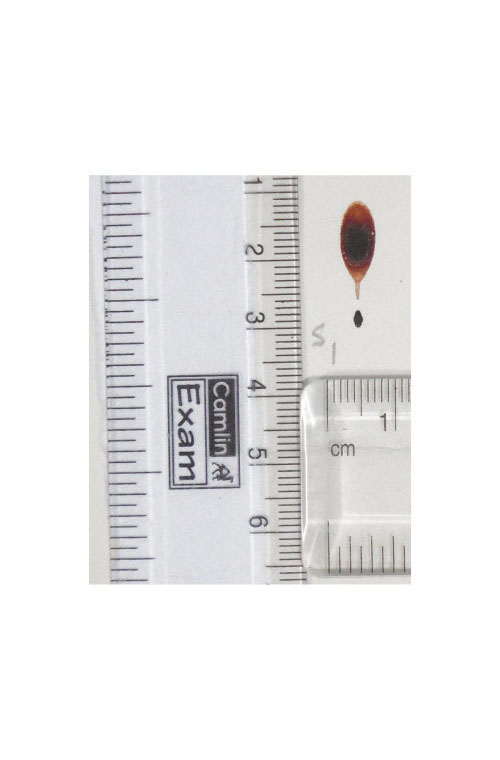

Supplement: Supplementary file 2 — Supplementary material [file mmc2.zip › Bloodstain_dataset/Heparin/DSCN1026.jpg]

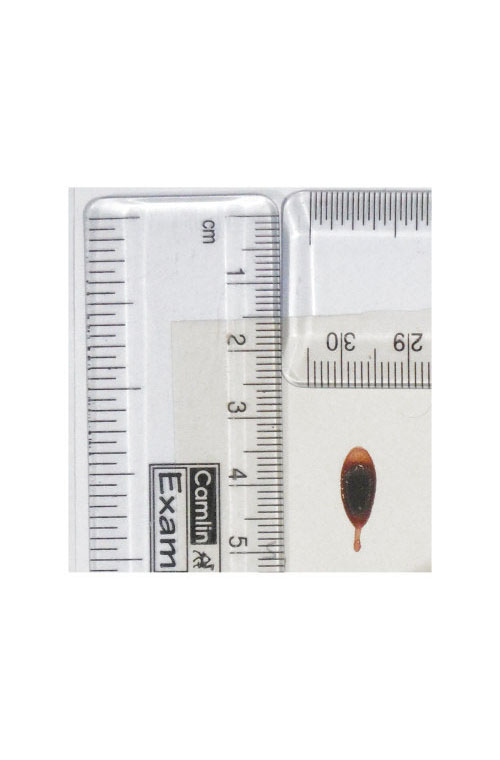

Supplement: Supplementary file 2 — Supplementary material [file mmc2.zip › Bloodstain_dataset/Heparin/DSCN1029.jpg]

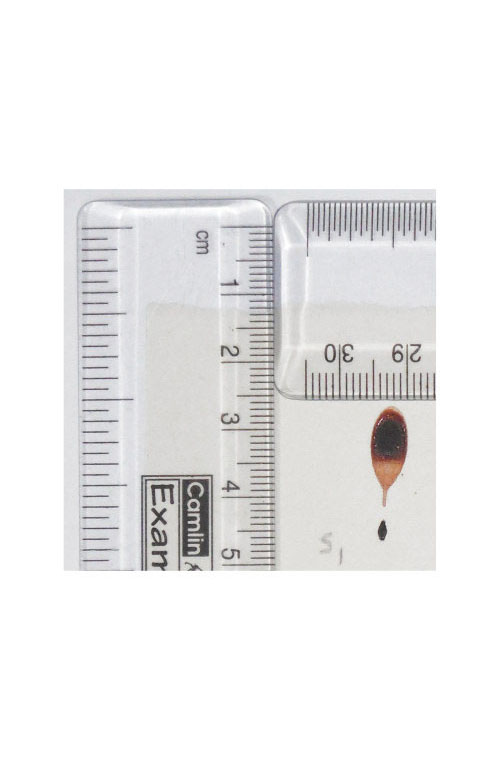

Supplement: Supplementary file 2 — Supplementary material [file mmc2.zip › Bloodstain_dataset/Heparin/DSCN1034.jpg]

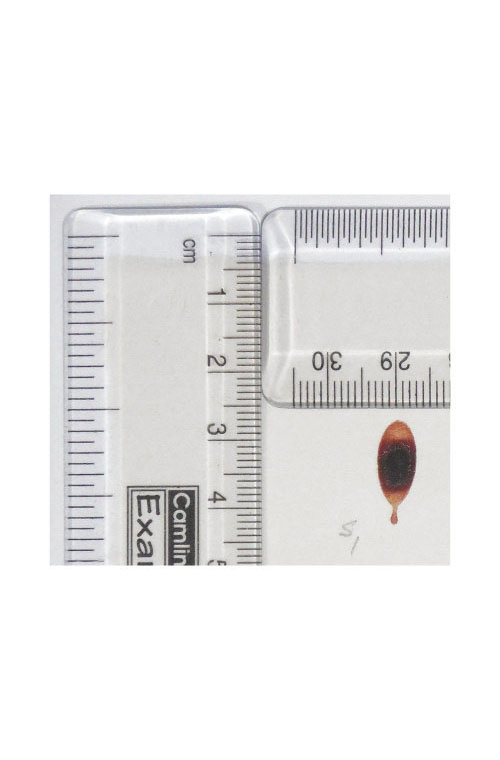

Supplement: Supplementary file 2 — Supplementary material [file mmc2.zip › Bloodstain_dataset/Heparin/DSCN1039.jpg]

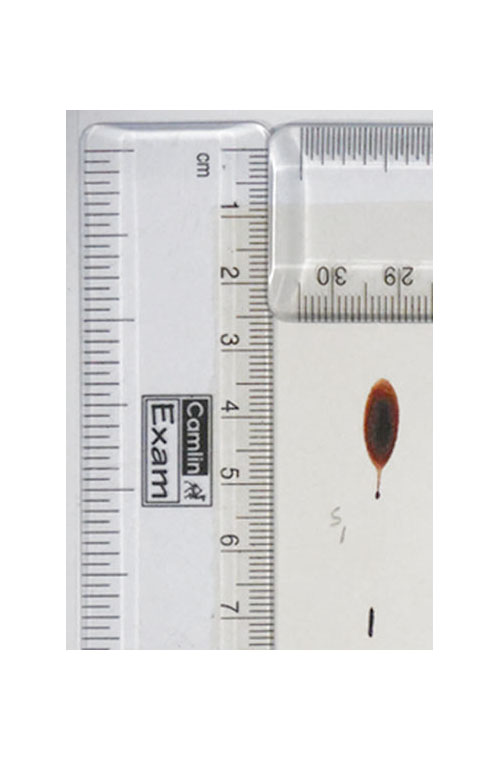

Supplement: Supplementary file 2 — Supplementary material [file mmc2.zip › Bloodstain_dataset/Heparin/DSCN1044.jpg]

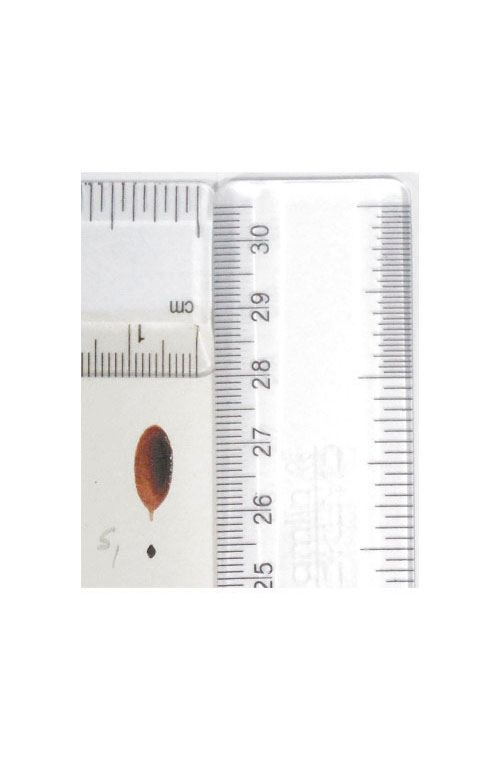

Supplement: Supplementary file 2 — Supplementary material [file mmc2.zip › Bloodstain_dataset/Heparin/DSCN1048.jpg]

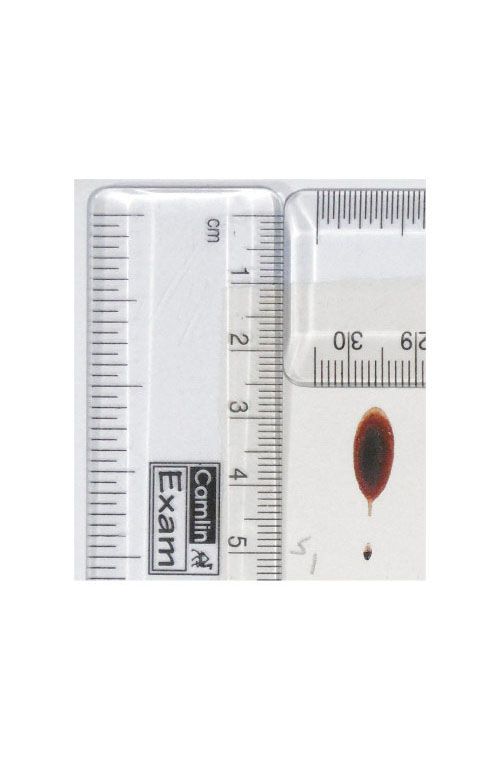

Supplement: Supplementary file 2 — Supplementary material [file mmc2.zip › Bloodstain_dataset/Heparin/DSCN1052.jpg]

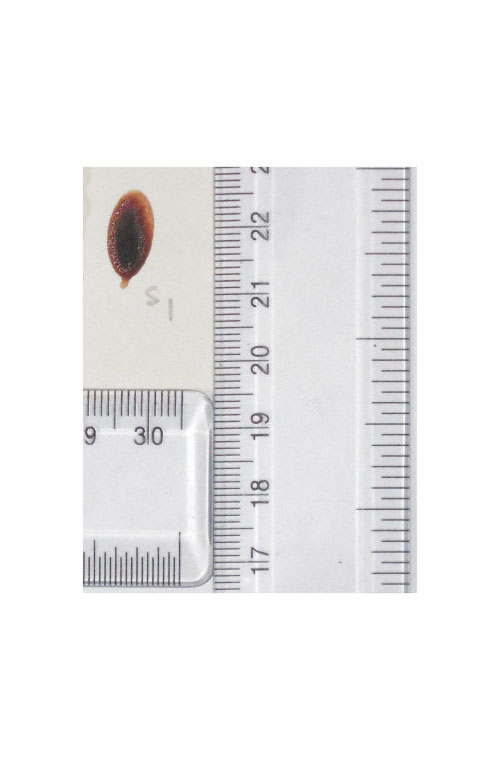

Supplement: Supplementary file 2 — Supplementary material [file mmc2.zip › Bloodstain_dataset/Heparin/DSCN1056.jpg]

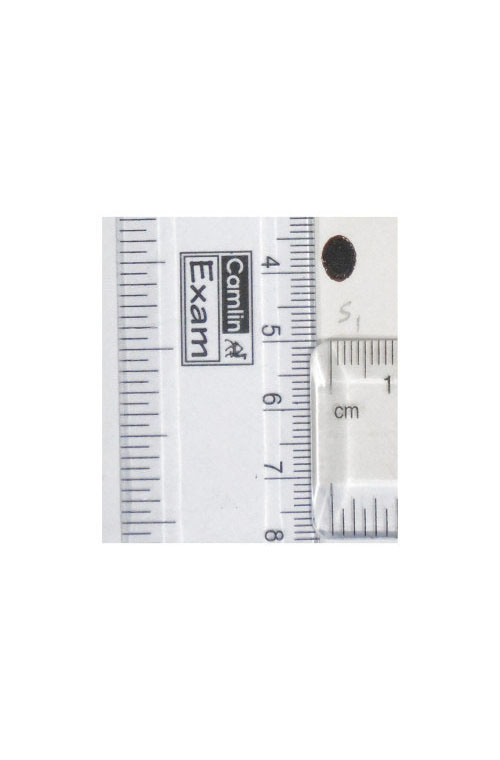

Supplement: Supplementary file 2 — Supplementary material [file mmc2.zip › Bloodstain_dataset/Heparin/DSCN1061.jpg]

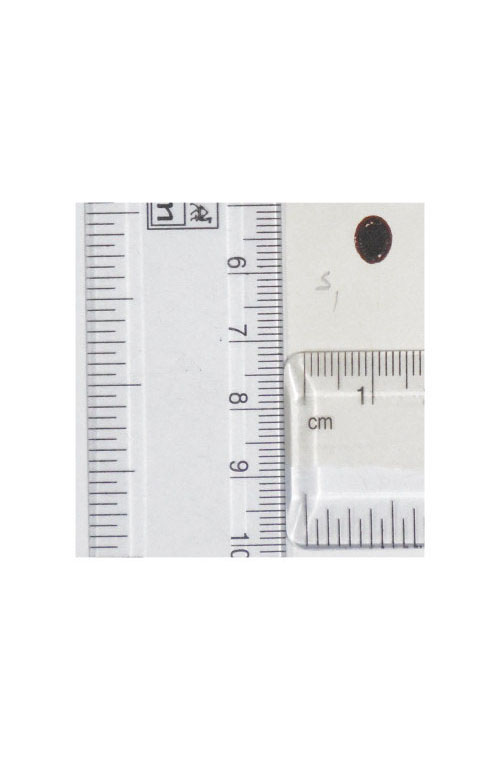

Supplement: Supplementary file 2 — Supplementary material [file mmc2.zip › Bloodstain_dataset/Heparin/DSCN1067.jpg]

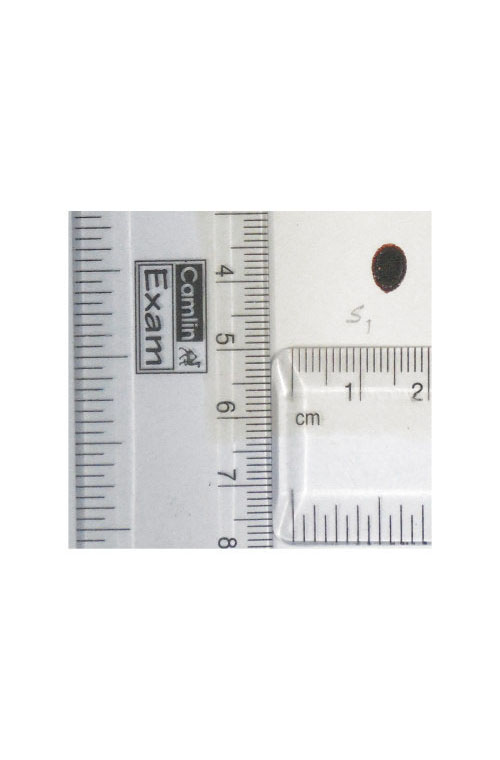

Supplement: Supplementary file 2 — Supplementary material [file mmc2.zip › Bloodstain_dataset/Heparin/DSCN1069.jpg]

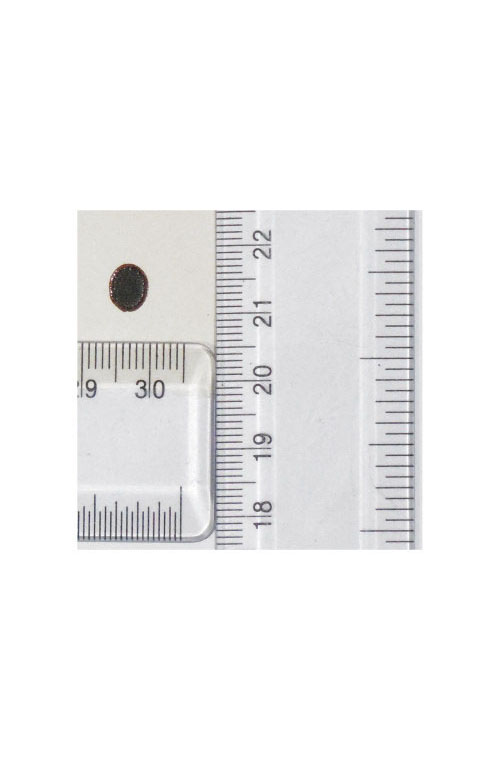

Supplement: Supplementary file 2 — Supplementary material [file mmc2.zip › Bloodstain_dataset/Heparin/DSCN1074.jpg]

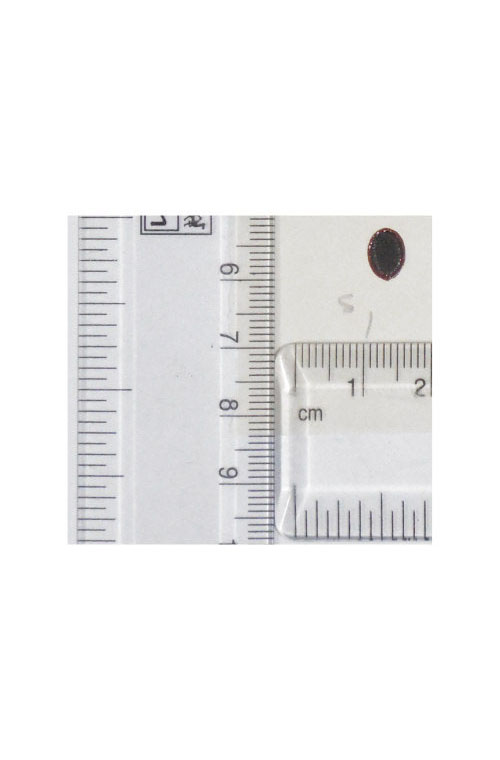

Supplement: Supplementary file 2 — Supplementary material [file mmc2.zip › Bloodstain_dataset/Heparin/DSCN1076.jpg]

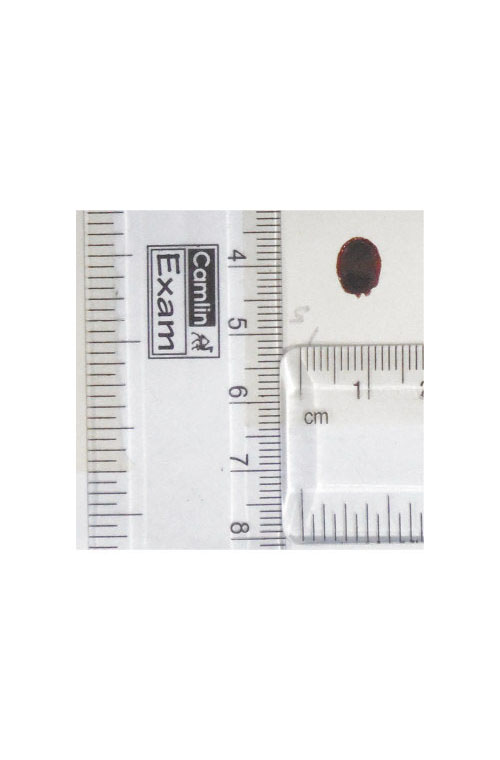

Supplement: Supplementary file 2 — Supplementary material [file mmc2.zip › Bloodstain_dataset/Heparin/DSCN1084.jpg]

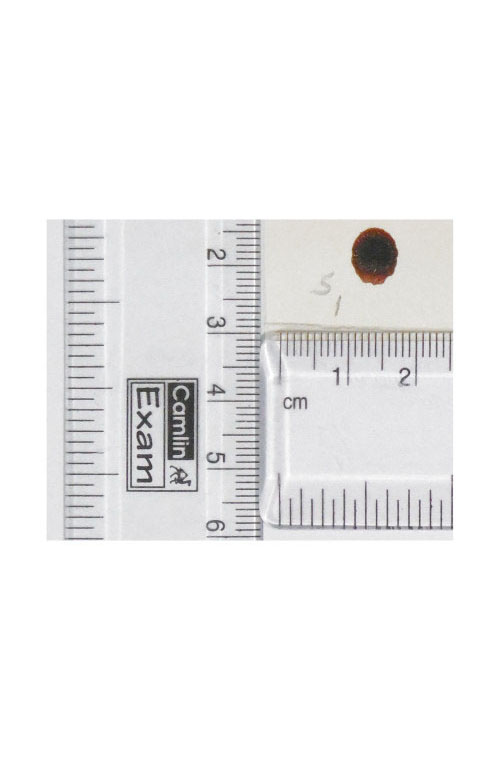

Supplement: Supplementary file 2 — Supplementary material [file mmc2.zip › Bloodstain_dataset/Heparin/DSCN1087.jpg]

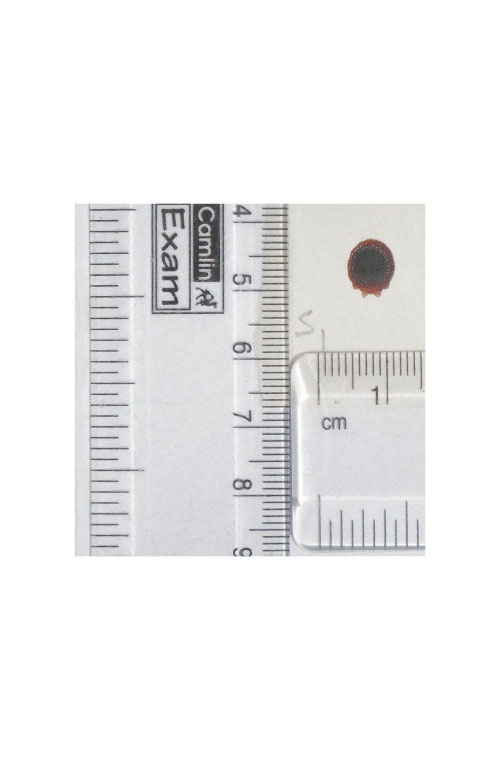

Supplement: Supplementary file 2 — Supplementary material [file mmc2.zip › Bloodstain_dataset/Heparin/DSCN1091.jpg]

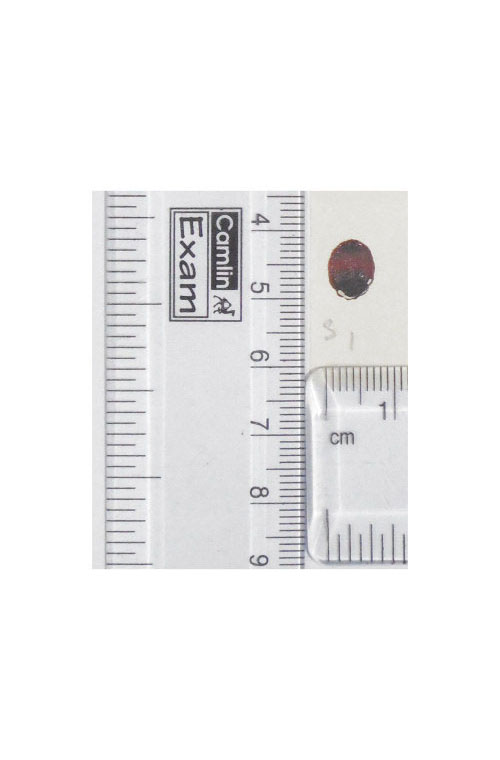

Supplement: Supplementary file 2 — Supplementary material [file mmc2.zip › Bloodstain_dataset/Heparin/DSCN1094.jpg]

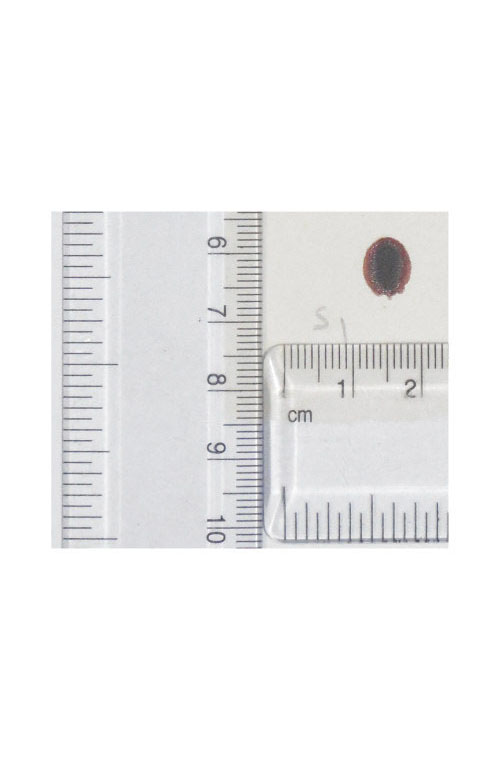

Supplement: Supplementary file 2 — Supplementary material [file mmc2.zip › Bloodstain_dataset/Heparin/DSCN1097.jpg]

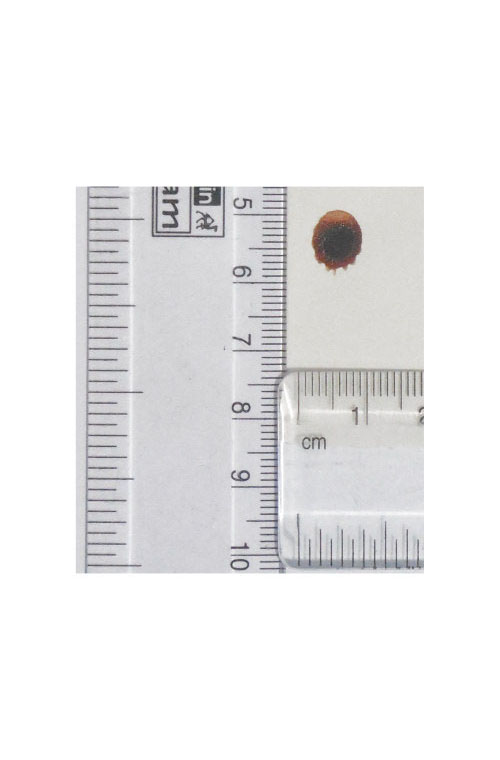

Supplement: Supplementary file 2 — Supplementary material [file mmc2.zip › Bloodstain_dataset/Heparin/DSCN1102.jpg]

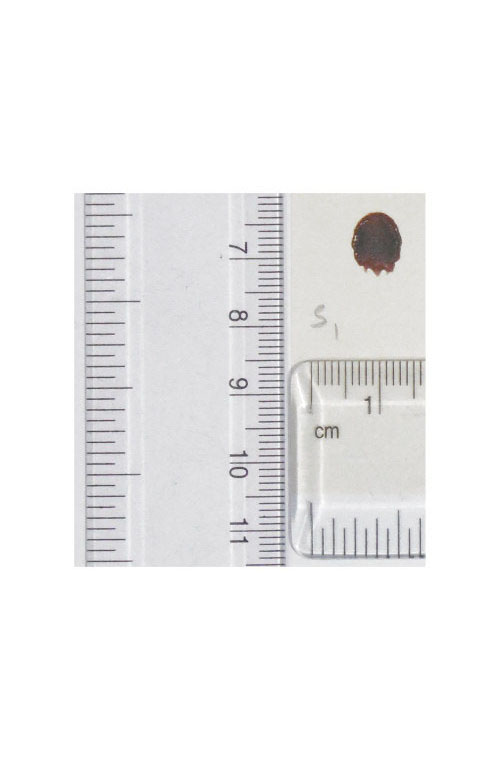

Supplement: Supplementary file 2 — Supplementary material [file mmc2.zip › Bloodstain_dataset/Heparin/DSCN1110.jpg]

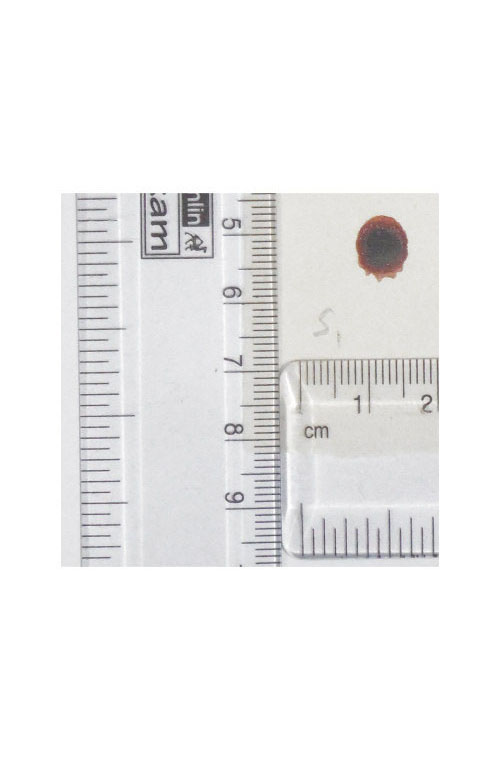

Supplement: Supplementary file 2 — Supplementary material [file mmc2.zip › Bloodstain_dataset/Heparin/DSCN1113.jpg]

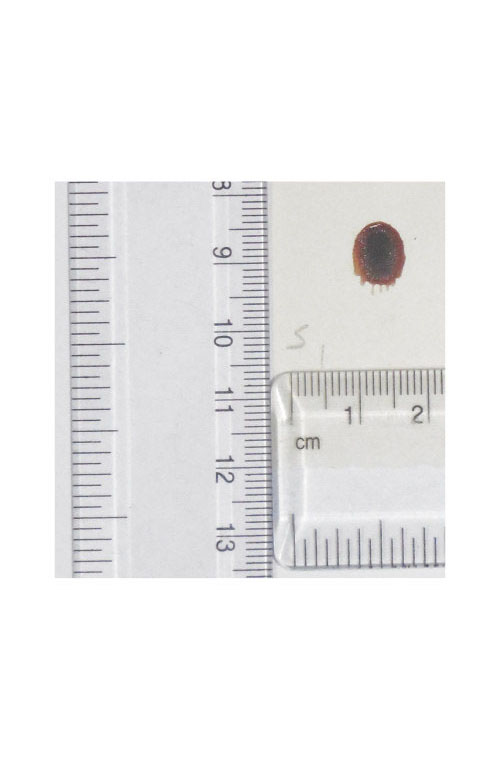

Supplement: Supplementary file 2 — Supplementary material [file mmc2.zip › Bloodstain_dataset/Heparin/DSCN1116.jpg]

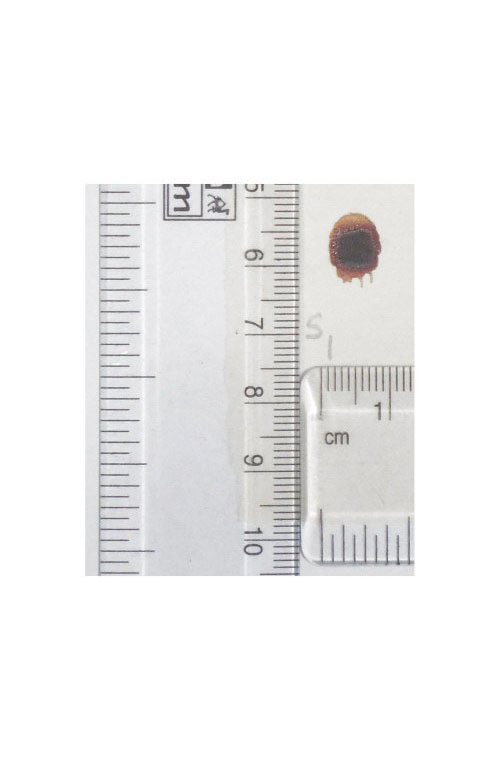

Supplement: Supplementary file 2 — Supplementary material [file mmc2.zip › Bloodstain_dataset/Heparin/DSCN1121.jpg]

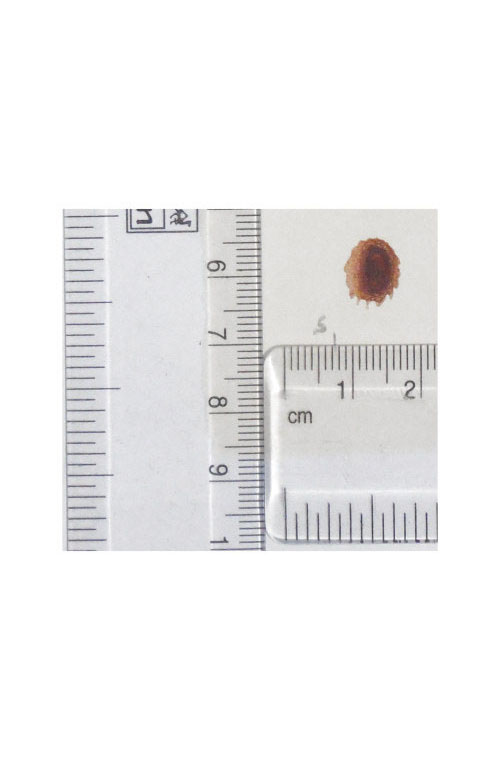

Supplement: Supplementary file 2 — Supplementary material [file mmc2.zip › Bloodstain_dataset/Subcutaneous/Syringe With Needle/DSCN0767.jpg]

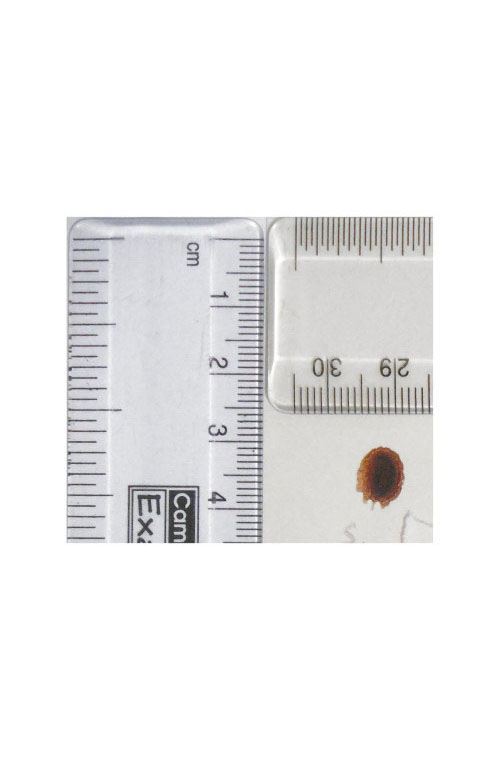

Supplement: Supplementary file 2 — Supplementary material [file mmc2.zip › Bloodstain_dataset/Subcutaneous/Syringe With Needle/DSCN0793.jpg]

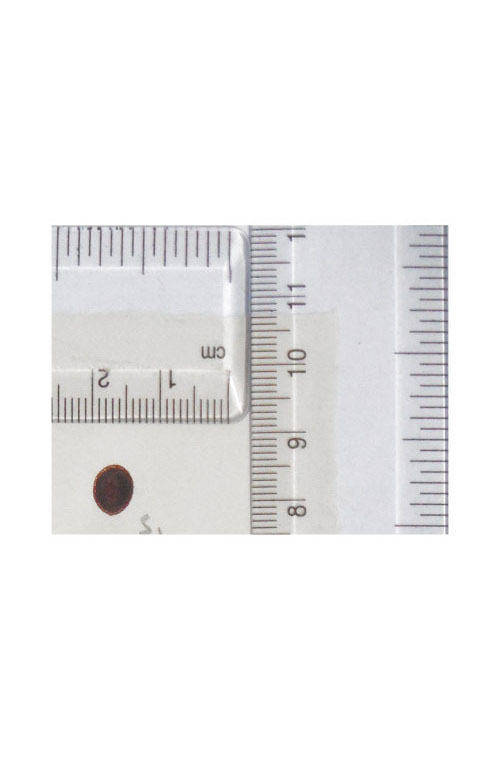

Supplement: Supplementary file 2 — Supplementary material [file mmc2.zip › Bloodstain_dataset/Subcutaneous/Syringe With Needle/DSCN0816.jpg]

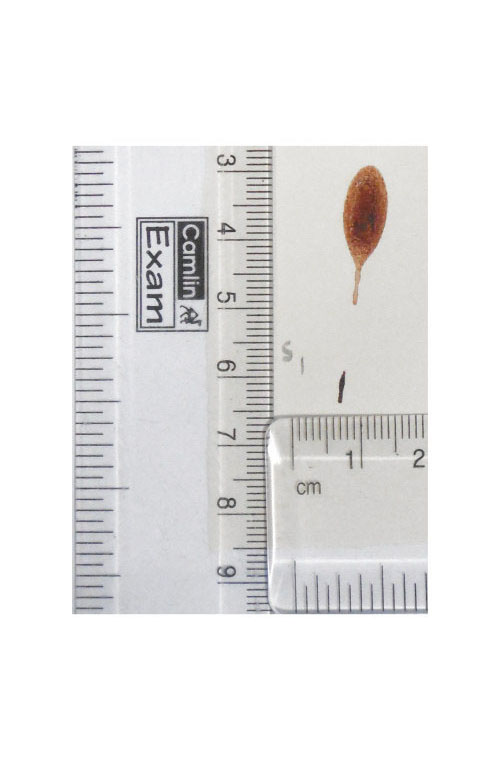

Supplement: Supplementary file 2 — Supplementary material [file mmc2.zip › Bloodstain_dataset/Subcutaneous/Syringe With Needle/DSCN0836.jpg]

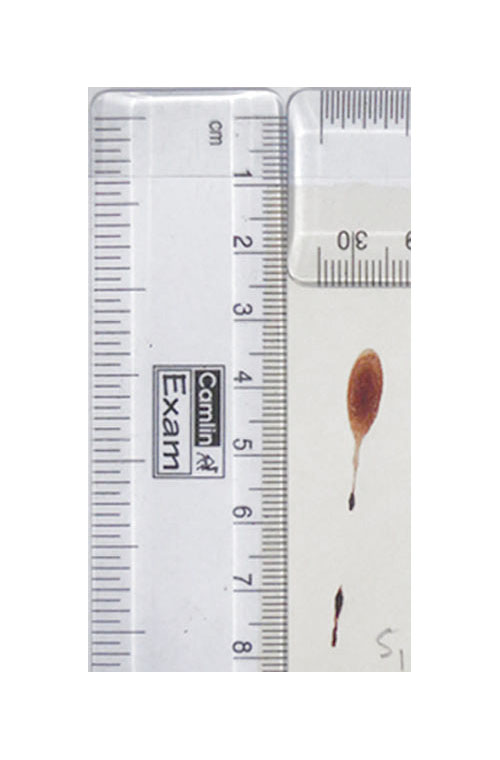

Supplement: Supplementary file 2 — Supplementary material [file mmc2.zip › Bloodstain_dataset/Subcutaneous/Syringe With Needle/DSCN0858.jpg]

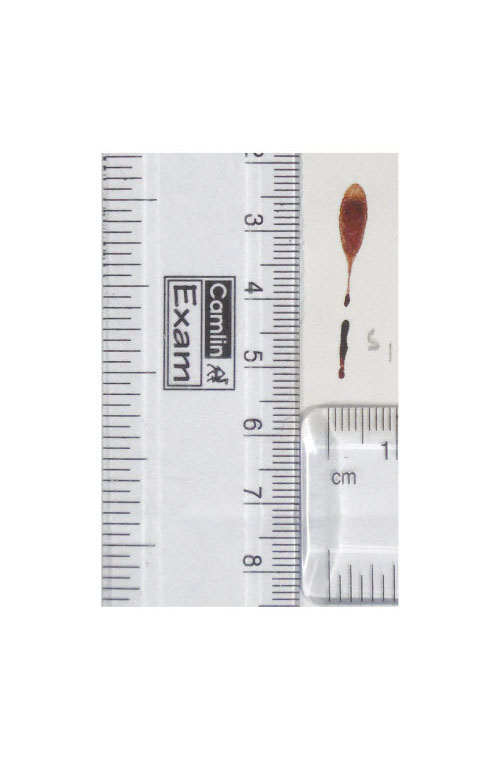

Supplement: Supplementary file 2 — Supplementary material [file mmc2.zip › Bloodstain_dataset/Subcutaneous/Syringe With Needle/DSCN0880.jpg]

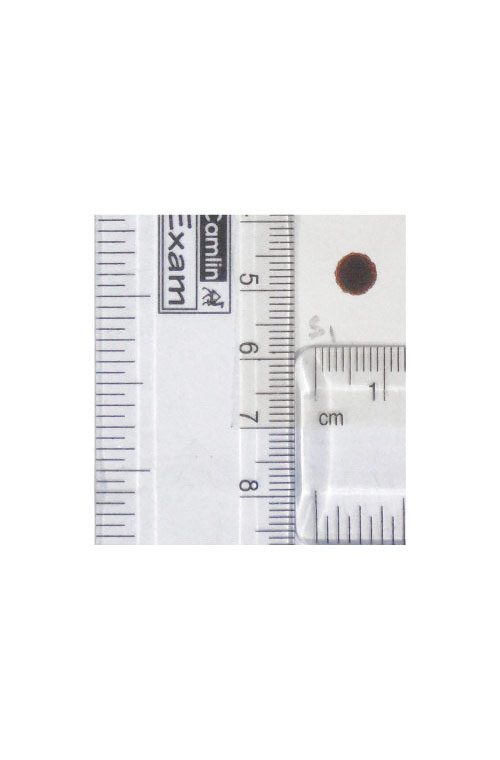

Supplement: Supplementary file 2 — Supplementary material [file mmc2.zip › Bloodstain_dataset/Subcutaneous/Syringe With Needle/DSCN0934.jpg]

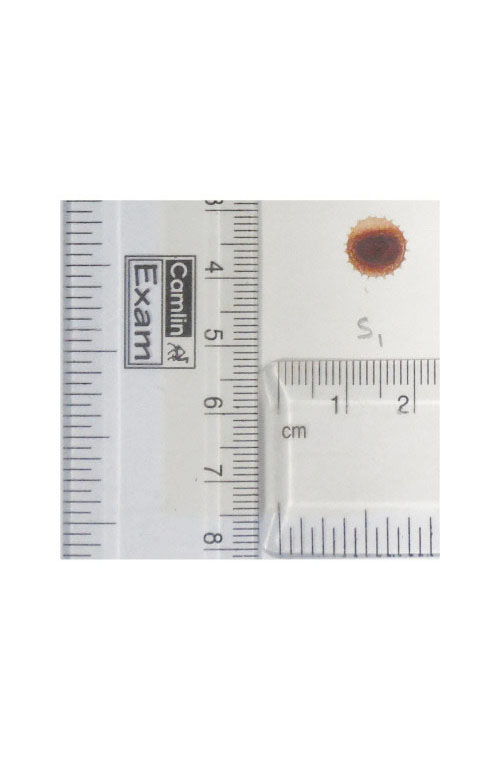

Supplement: Supplementary file 2 — Supplementary material [file mmc2.zip › Bloodstain_dataset/Subcutaneous/Syringe With Needle/DSCN0957.jpg]

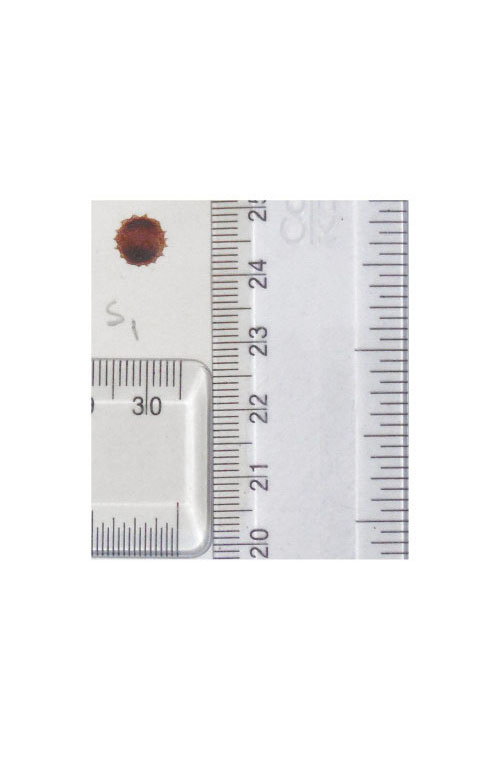

Supplement: Supplementary file 2 — Supplementary material [file mmc2.zip › Bloodstain_dataset/Subcutaneous/Syringe With Needle/DSCN0977.jpg]

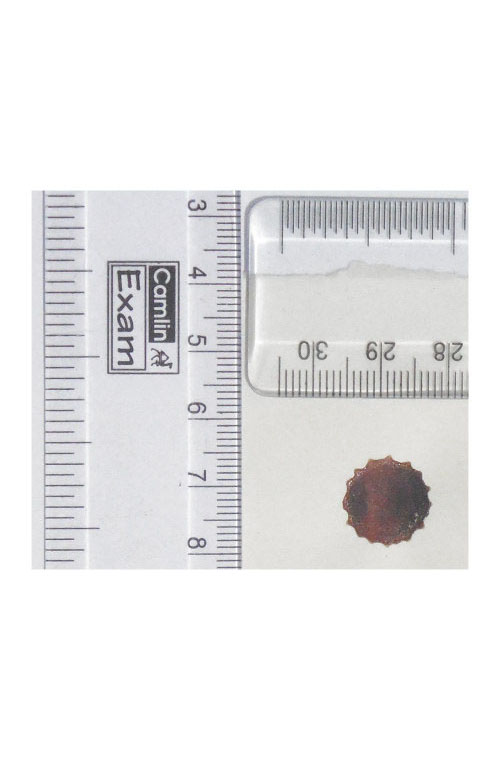

Supplement: Supplementary file 2 — Supplementary material [file mmc2.zip › Bloodstain_dataset/Subcutaneous/Syringe Without Needle/DSCN1125.jpg]

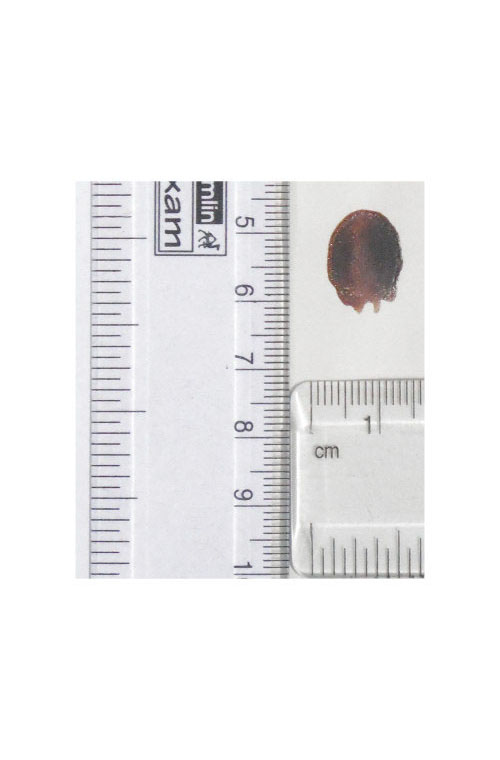

Supplement: Supplementary file 2 — Supplementary material [file mmc2.zip › Bloodstain_dataset/Subcutaneous/Syringe Without Needle/DSCN1129.jpg]

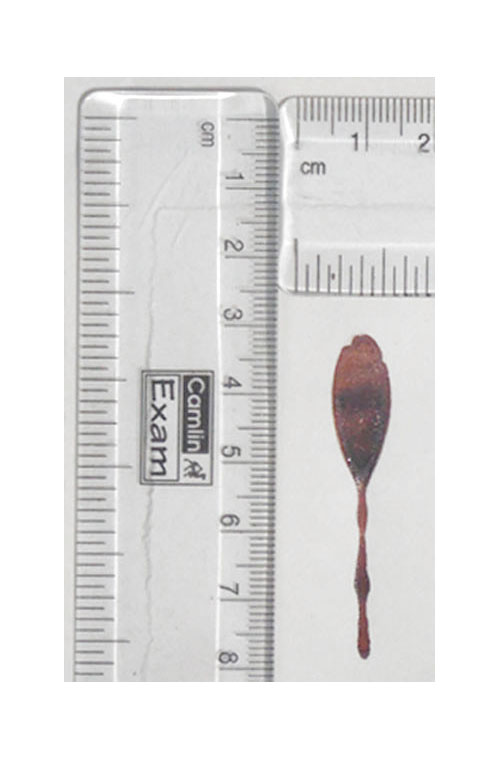

Supplement: Supplementary file 2 — Supplementary material [file mmc2.zip › Bloodstain_dataset/Subcutaneous/Syringe Without Needle/DSCN1132.jpg]

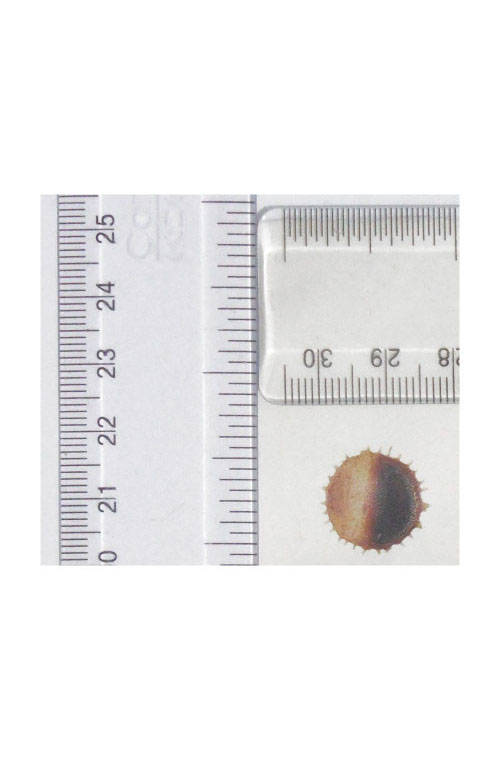

Supplement: Supplementary file 2 — Supplementary material [file mmc2.zip › Bloodstain_dataset/Subcutaneous/Syringe Without Needle/DSCN1137.jpg]

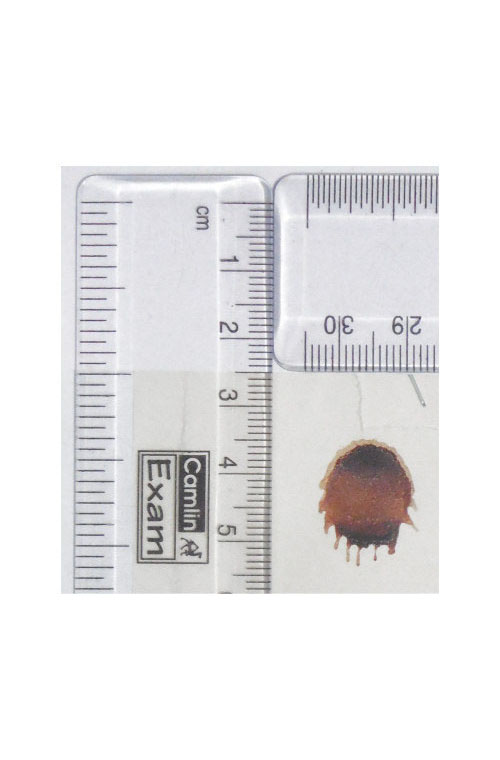

Supplement: Supplementary file 2 — Supplementary material [file mmc2.zip › Bloodstain_dataset/Subcutaneous/Syringe Without Needle/DSCN1140.jpg]

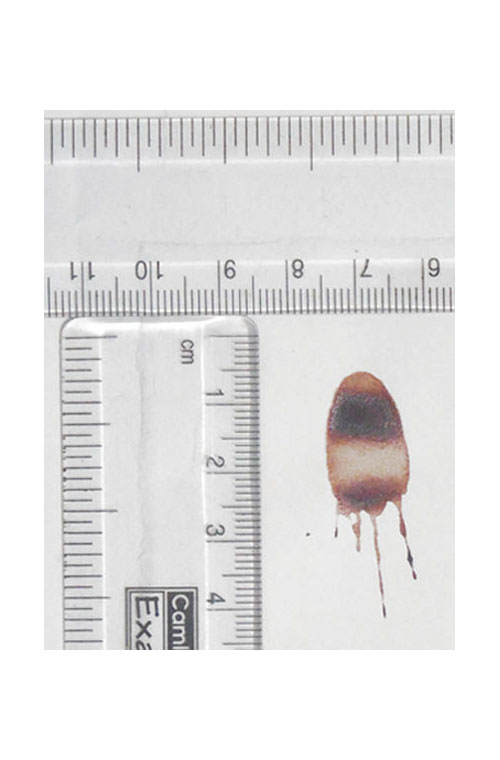

Supplement: Supplementary file 2 — Supplementary material [file mmc2.zip › Bloodstain_dataset/Subcutaneous/Syringe Without Needle/DSCN1144.jpg]

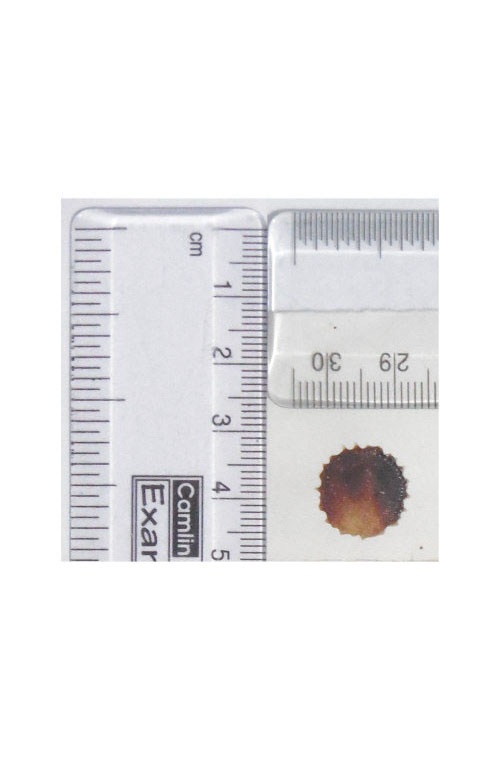

Supplement: Supplementary file 2 — Supplementary material [file mmc2.zip › Bloodstain_dataset/Subcutaneous/Syringe Without Needle/DSCN1146.jpg]

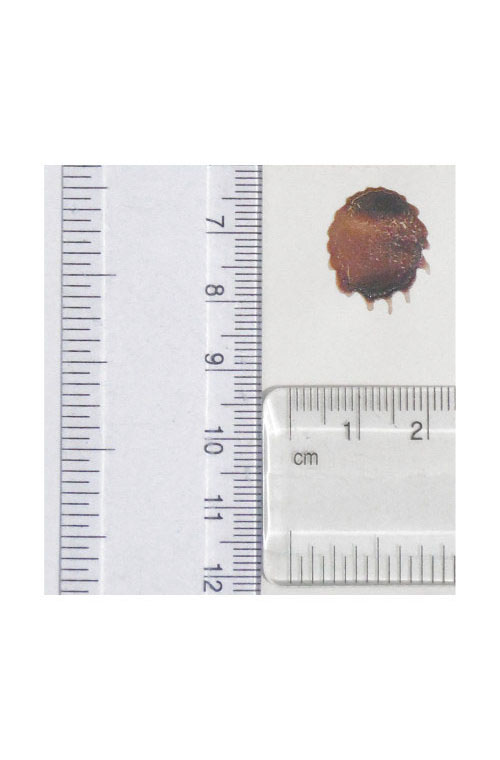

Supplement: Supplementary file 2 — Supplementary material [file mmc2.zip › Bloodstain_dataset/Subcutaneous/Syringe Without Needle/DSCN1148.jpg]

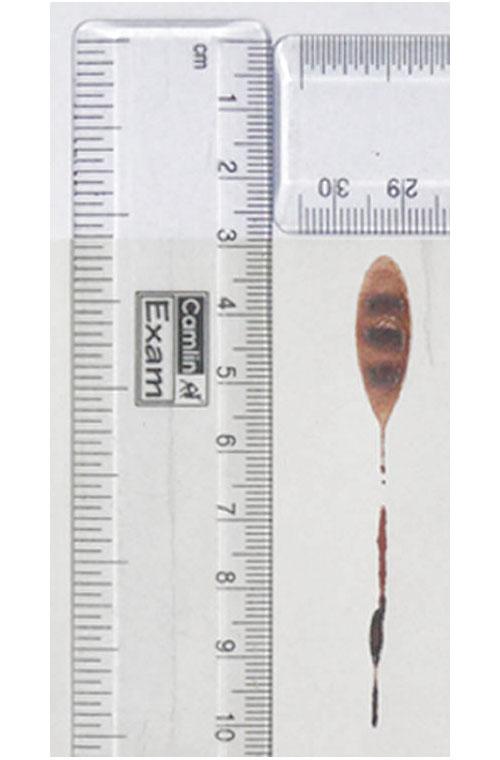

Supplement: Supplementary file 2 — Supplementary material [file mmc2.zip › Bloodstain_dataset/Subcutaneous/Syringe Without Needle/DSCN1151.jpg]

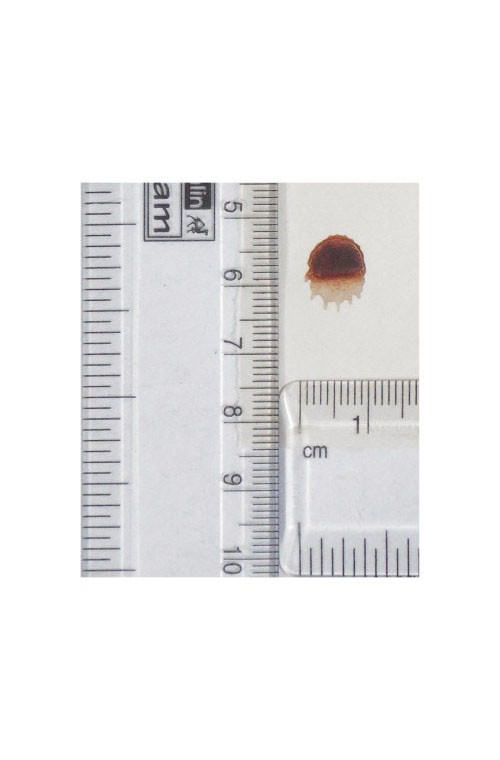

Supplement: Supplementary file 2 — Supplementary material [file mmc2.zip › Bloodstain_dataset/Warfarin/DSCN0770.jpg]

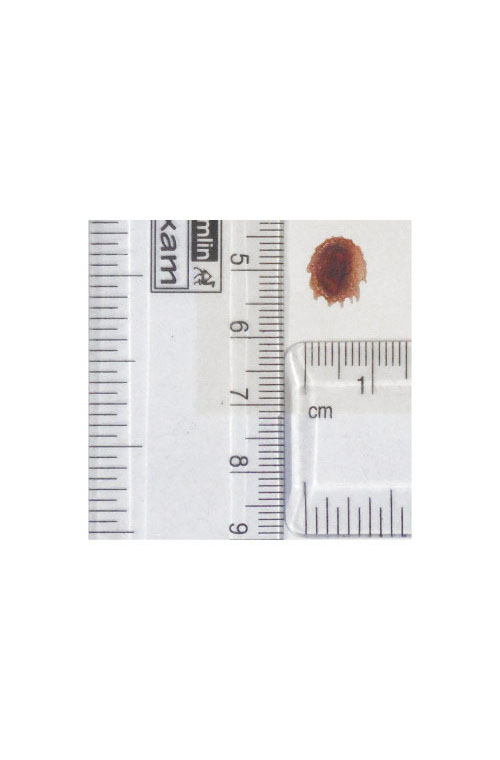

Supplement: Supplementary file 2 — Supplementary material [file mmc2.zip › Bloodstain_dataset/Warfarin/DSCN0773.jpg]

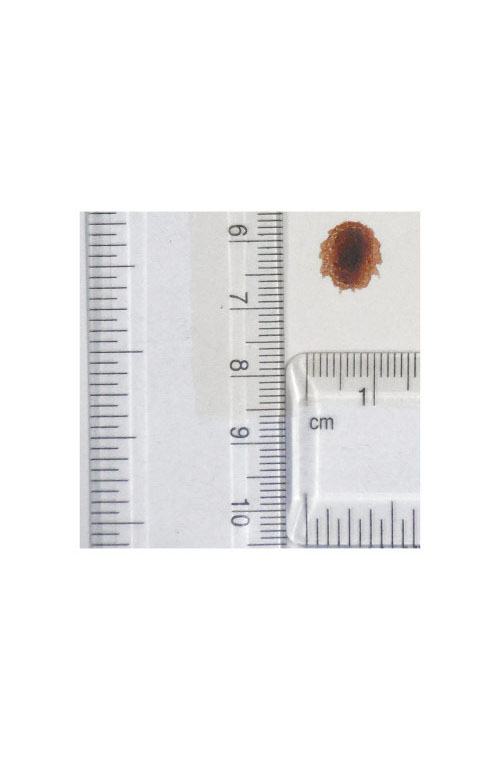

Supplement: Supplementary file 2 — Supplementary material [file mmc2.zip › Bloodstain_dataset/Warfarin/DSCN0777.jpg]

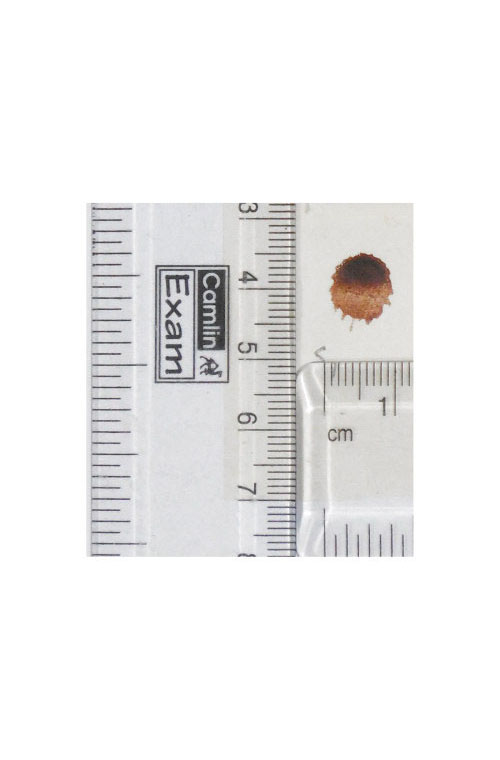

Supplement: Supplementary file 2 — Supplementary material [file mmc2.zip › Bloodstain_dataset/Warfarin/DSCN0786.jpg]

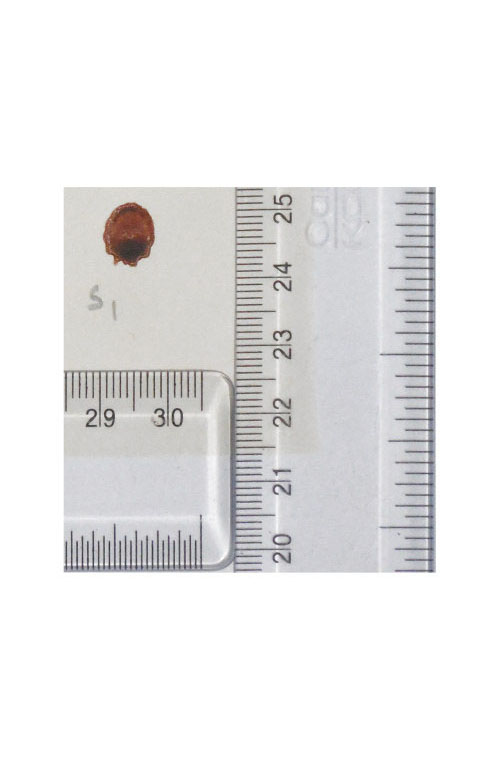

Supplement: Supplementary file 2 — Supplementary material [file mmc2.zip › Bloodstain_dataset/Warfarin/DSCN0798.jpg]

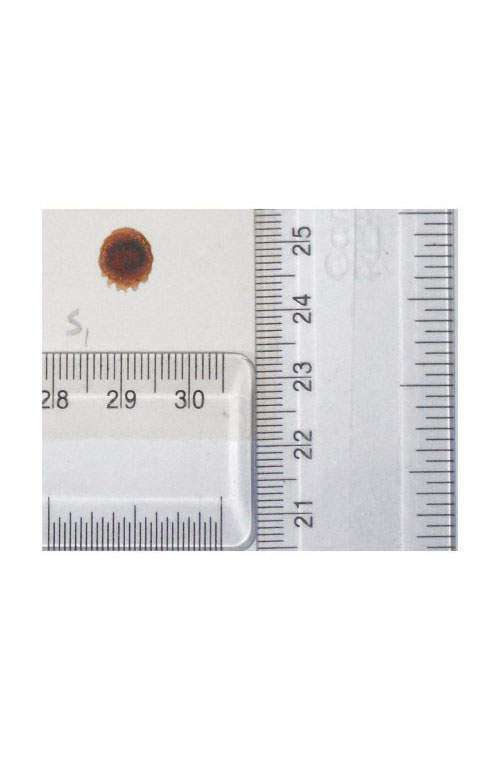

Supplement: Supplementary file 2 — Supplementary material [file mmc2.zip › Bloodstain_dataset/Warfarin/DSCN0802.jpg]

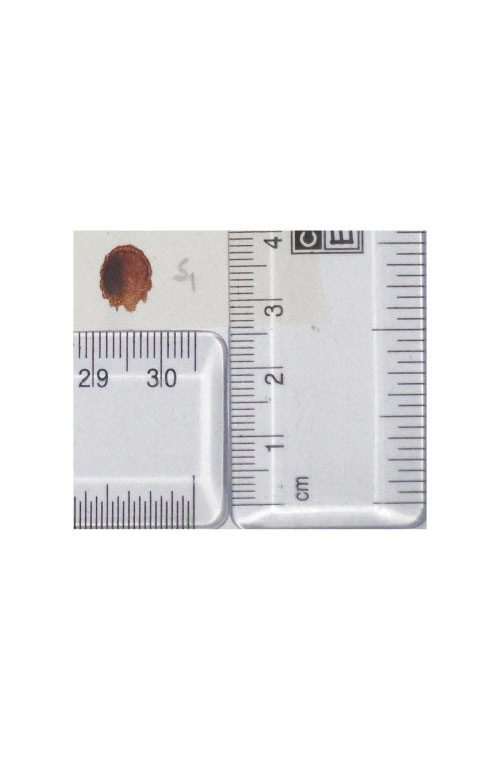

Supplement: Supplementary file 2 — Supplementary material [file mmc2.zip › Bloodstain_dataset/Warfarin/DSCN0807.jpg]

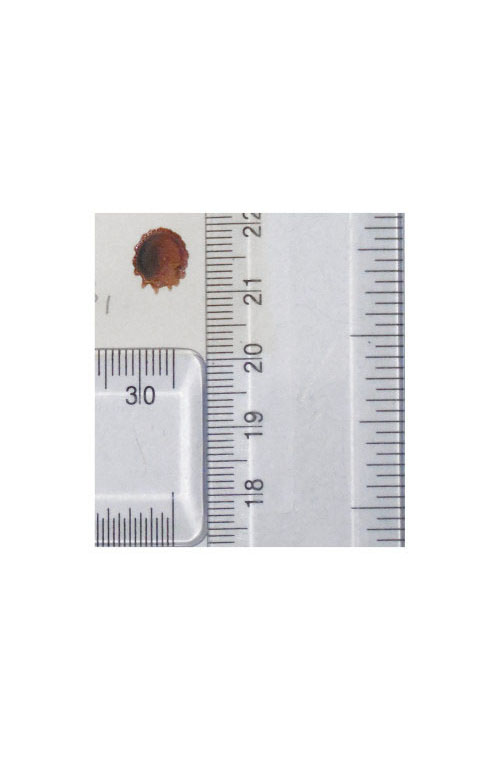

Supplement: Supplementary file 2 — Supplementary material [file mmc2.zip › Bloodstain_dataset/Warfarin/DSCN0811.jpg]

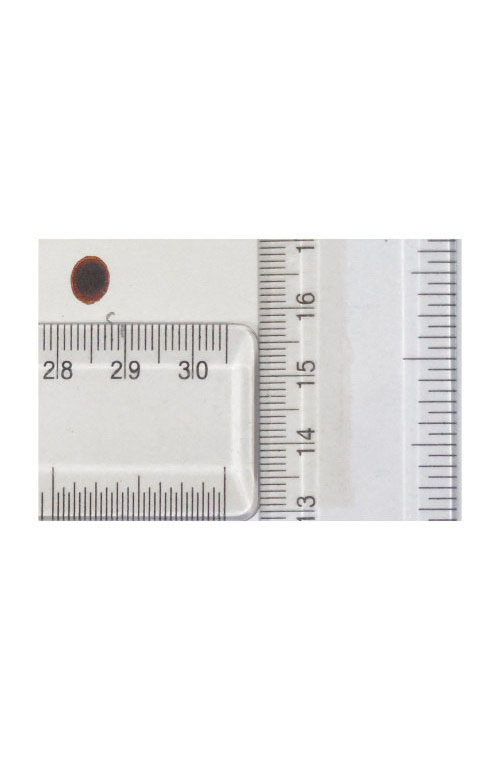

Supplement: Supplementary file 2 — Supplementary material [file mmc2.zip › Bloodstain_dataset/Warfarin/DSCN0818.jpg]

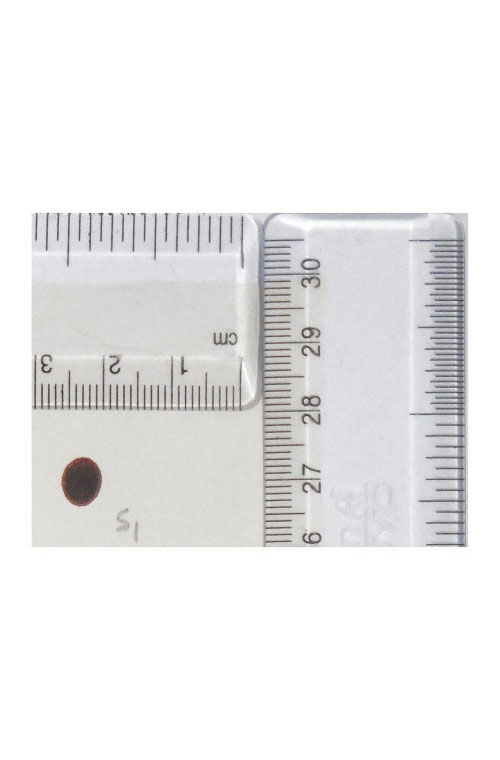

Supplement: Supplementary file 2 — Supplementary material [file mmc2.zip › Bloodstain_dataset/Warfarin/DSCN0823.jpg]

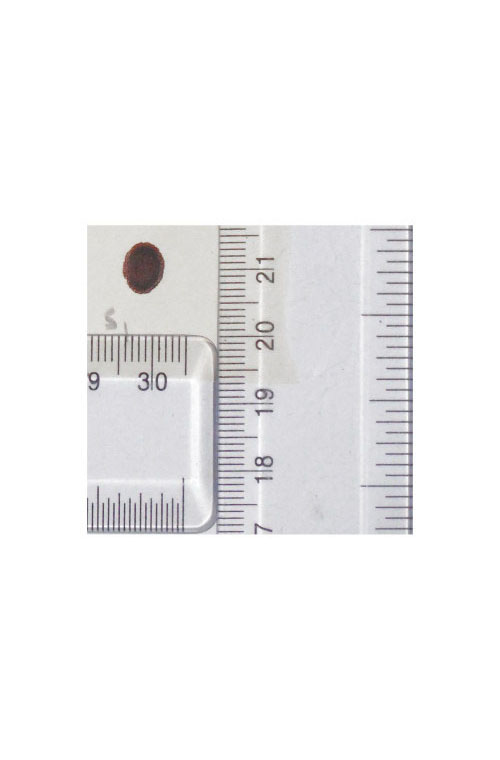

Supplement: Supplementary file 2 — Supplementary material [file mmc2.zip › Bloodstain_dataset/Warfarin/DSCN0824.jpg]

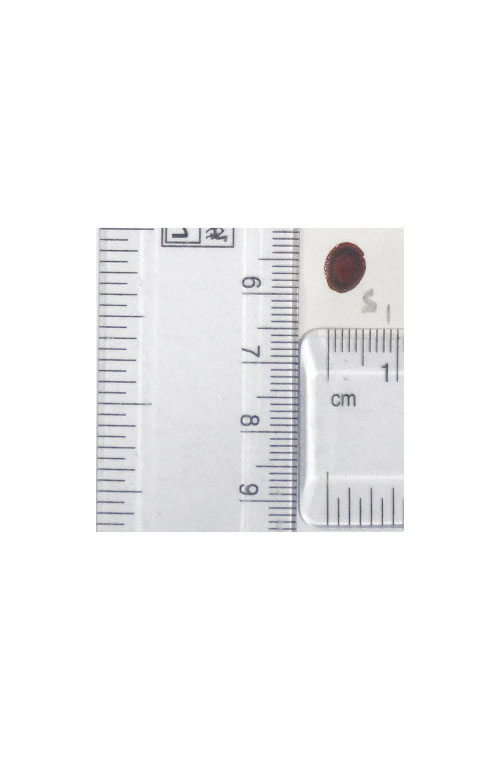

Supplement: Supplementary file 2 — Supplementary material [file mmc2.zip › Bloodstain_dataset/Warfarin/DSCN0827.jpg]

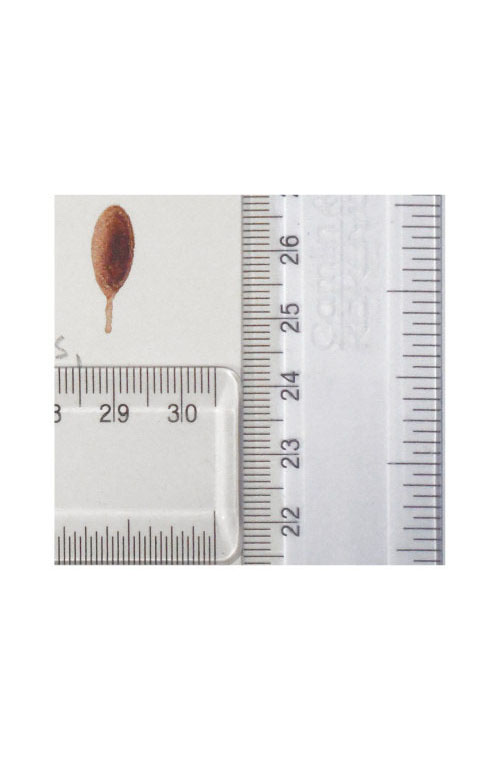

Supplement: Supplementary file 2 — Supplementary material [file mmc2.zip › Bloodstain_dataset/Warfarin/DSCN0841.jpg]

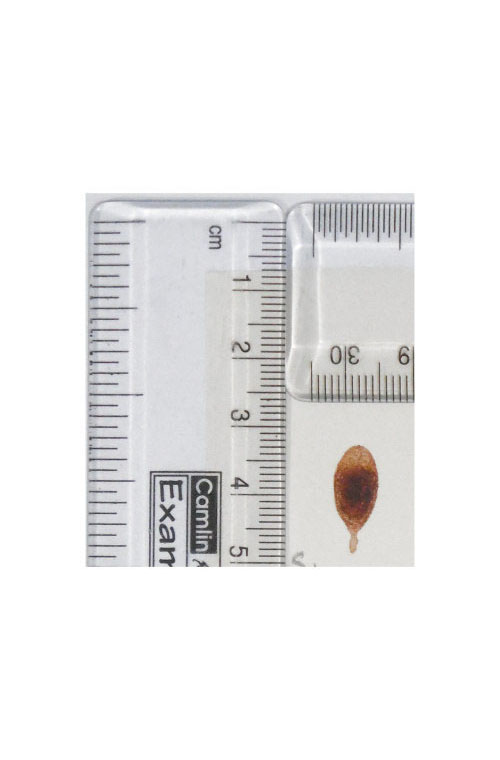

Supplement: Supplementary file 2 — Supplementary material [file mmc2.zip › Bloodstain_dataset/Warfarin/DSCN0844.jpg]

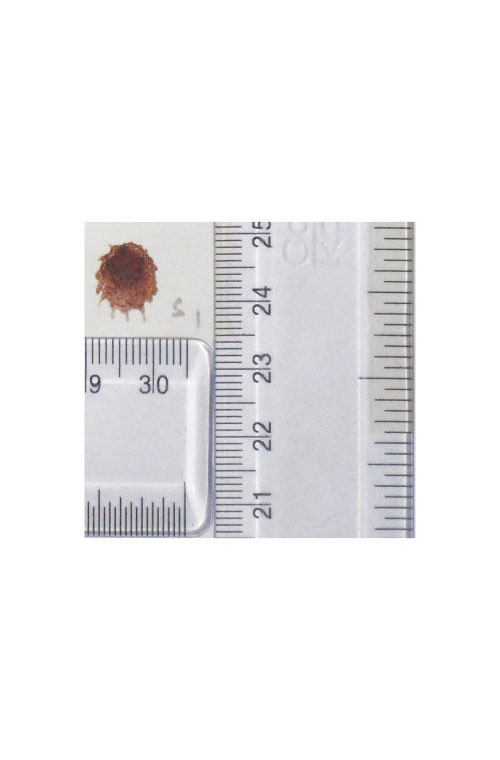

Supplement: Supplementary file 2 — Supplementary material [file mmc2.zip › Bloodstain_dataset/Warfarin/DSCN0849.jpg]

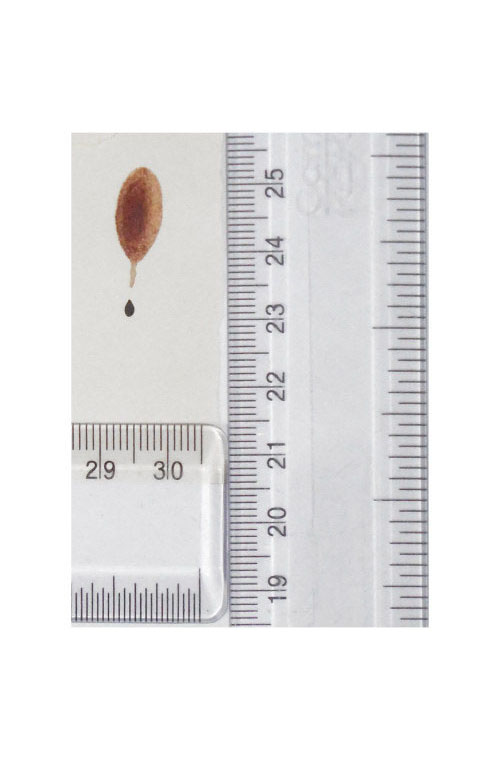

Supplement: Supplementary file 2 — Supplementary material [file mmc2.zip › Bloodstain_dataset/Warfarin/DSCN0853.jpg]

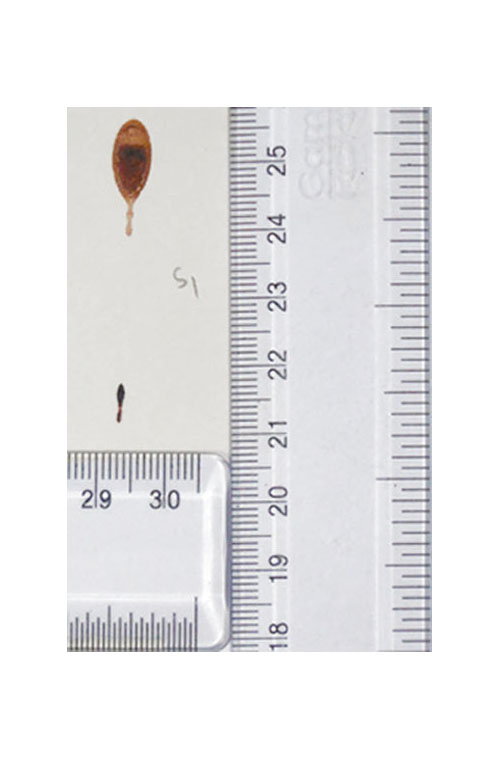

Supplement: Supplementary file 2 — Supplementary material [file mmc2.zip › Bloodstain_dataset/Warfarin/DSCN0862.jpg]

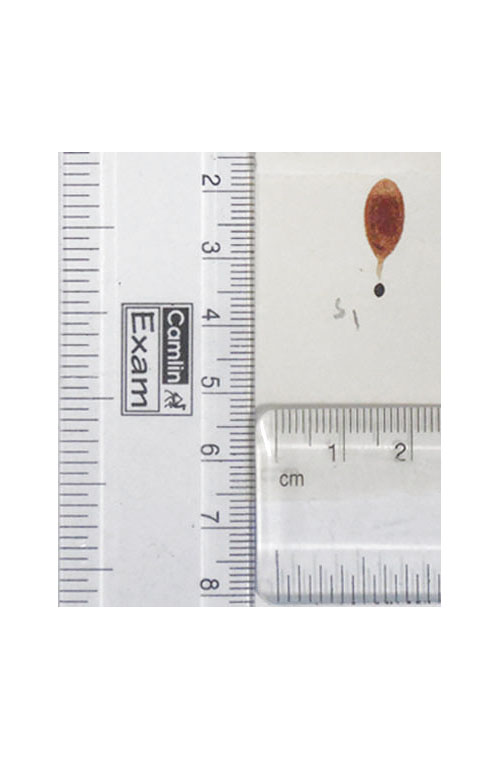

Supplement: Supplementary file 2 — Supplementary material [file mmc2.zip › Bloodstain_dataset/Warfarin/DSCN0866.jpg]

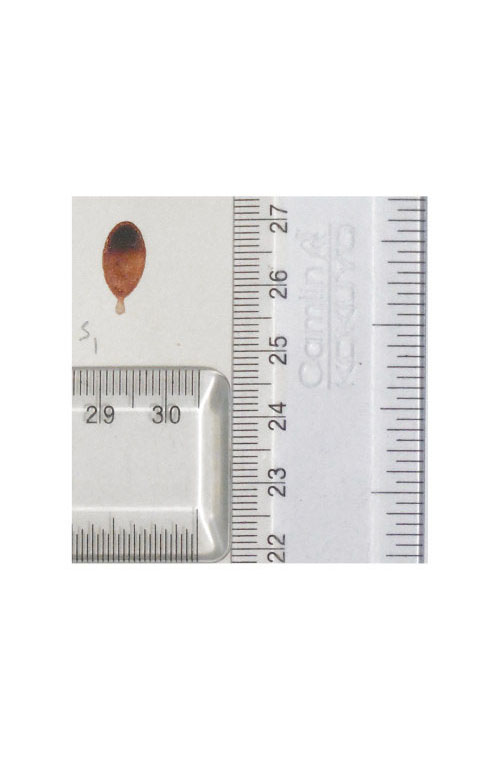

Supplement: Supplementary file 2 — Supplementary material [file mmc2.zip › Bloodstain_dataset/Warfarin/DSCN0869.jpg]
